# Supplementary material for: Associations between falls and other serious adverse events and antihypertensive medication in individuals with dementia: An observational cohort study
Source: PLoS Med. 2025 Sep 17;22(9):e1004731. doi: 10.1371/journal.pmed.1004731 (PMC12478963; doi:10.1371/journal.pmed.1004731)
Supplement: S2 Table — CPRD indicates Clinical Practice Research Datalink. (DOCX) [file pmed.1004731.s003.docx]

| **Supplementary Table S2. The CPRD GOLD medical codes used to define dementia** | | | |
| --- | --- | --- | --- |
| **Types of dementia** | **medcode** | **Read code** | **Description** |
| **Alzheimer’s disease** | 16797 | F110000 | Alzheimer's disease with early onset |
|  | 61528 | Eu00013 | Alzheimer's disease type 2 |
|  | 11379 | Eu00112 | Senile dementia, Alzheimer's type |
|  | 59122 | Fyu3000 | Other Alzheimer's disease |
|  | 32057 | F110100 | Alzheimer's disease with late onset |
|  | 60059 | Eu00012 | Primary degen dementia, Alzheimer's type, presenile onset |
|  | 25704 | Eu00011 | Presenile dementia, Alzheimer's type |
|  | 46762 | Eu00111 | Alzheimer's disease type 1 |
|  | 8195 | Eu00z11 | Alzheimer's dementia unspec |
|  | 1917 | F110.00 | Alzheimer's disease |
|  | 28626 | 13Y7.00 | Alzheimer's disease society member |
|  | 38678 | Eu00100 | Dementia in Alzheimer's disease with late onset |
|  | 49263 | Eu00000 | Dementia in Alzheimer's disease with early onset |
|  | 43346 | Eu00113 | Primary degen dementia of Alzheimer's type, senile onset |
|  | 7664 | Eu00.00 | Dementia in Alzheimer's disease |
|  | 29386 | Eu00z00 | Dementia in Alzheimer's disease, unspecified |
|  | 111349 | 8T0Y.00 | Referral to Alzheimer's Society |
|  | 30706 | Eu00200 | Dementia in Alzheimer's dis, atypical or mixed type |
| **Vascular dementia** | 31016 | Eu01300 | Mixed cortical and subcortical vascular dementia |
|  | 43292 | E004300 | Arteriosclerotic dementia with depression |
|  | 6578 | Eu01.00 | Vascular dementia |
|  | 19393 | Eu01z00 | Vascular dementia, unspecified |
|  | 19477 | E004.00 | Arteriosclerotic dementia |
|  | 8634 | E004.11 | Multi infarct dementia |
|  | 8934 | Eu01200 | Subcortical vascular dementia |
|  | 55313 | Eu01y00 | Other vascular dementia |
|  | 43089 | E004000 | Uncomplicated arteriosclerotic dementia |
|  | 42279 | E004z00 | Arteriosclerotic dementia NOS |
|  | 11175 | Eu01100 | Multi-infarct dementia |
|  | 55467 | E004200 | Arteriosclerotic dementia with paranoia |
|  | 56912 | E004100 | Arteriosclerotic dementia with delirium |
|  | 46488 | Eu01000 | Vascular dementia of acute onset |
|  | 9565 | Eu01.11 | Arteriosclerotic dementia |
|  | 54744 | F11x200 | Cerebral degeneration due to cerebrovascular disease |
| **Other types of dementia** | 25386 | E041.00 | Dementia in conditions EC |
|  | 27677 | E001300 | Presenile dementia with depression |
|  | 33707 | E00..00 | Senile and presenile organic psychotic conditions |
|  | 104155 | 1JA2.00 | Suspected dementia |
|  | 103445 | 8Hla.00 | Referral to dementia care advisor |
|  | 21887 | E002100 | Senile dementia with depression |
|  | 108268 | 8CMG200 | Review of dementia advance care plan |
|  | 109731 | 8CMZ000 | Dementia care plan agreed |
|  | 3325 | R009.11 | Senile confusion |
|  | 55838 | Eu01111 | Predominantly cortical dementia |
|  | 63652 | F103000 | Cerebral degeneration in Hunter's disease |
|  | 106627 | 8T05.00 | Referral to dementia service |
|  | 44674 | E002.00 | Senile dementia with depressive or paranoid features |
|  | 4693 | Eu02z00 | Unspecified dementia |
|  | 12710 | 6AB..00 | Dementia annual review |
|  | 60680 | A941.00 | General paresis - neurosyphilis |
|  | 96860 | F11x900 | Cerebral degeneration in Parkinson's disease |
|  | 27342 | E012.11 | Alcoholic dementia NOS |
|  | 108228 | 8CSA.00 | Dementia advance care plan agreed |
|  | 109397 | 8T05000 | Referral to dementia support organization |
|  | 56288 | F10..00 | Cerebral degenerations usually manifest in childhood |
|  | 109786 | 8CMZ200 | Dementia care plan declined |
|  | 89037 | 9Ou4.00 | Dementia monitoring verbal invite |
|  | 31524 | F11yz00 | Other cerebral degeneration NOS |
|  | 108391 | 8IAe000 | Dementia advance care plan declined |
|  | 109047 | 8BPa.00 | Antipsychotic drug therapy for dementia |
|  | 34976 | F11y.00 | Other cerebral degeneration |
|  | 11670 | Eu10611 | Korsakov's psychosis, alcohol induced |
|  | 28402 | Eu02000 | Dementia in Pick's disease |
|  | 9509 | Eu02300 | Dementia in Parkinson's disease |
|  | 47555 | F11x000 | Cerebral degeneration due to alcoholism |
|  | 27935 | Eu02z15 | Senile psychosis NOS |
|  | 40805 | 9hD1.00 | Excepted from dementia quality indicators: Informed dissent |
|  | 83576 | 9Ou2.00 | Dementia monitoring second letter |
|  | 48501 | Eu02z11 | Presenile dementia NOS |
|  | 13570 | R200.11 | Senility |
|  | 5931 | 1461.00 | H/O: dementia |
|  | 27759 | Eu02z16 | Senile dementia, depressed or paranoid type |
|  | 11136 | F111.00 | Pick's disease |
|  | 102189 | ZR1T.00 | Arizona battery for communication disorders of dementia |
|  | 37698 | R20..00 | Senility, without mention of psychosis |
|  | 104534 | F118.00 | Frontotemporal degeneration |
|  | 26270 | Eu02500 | Lewy body dementia |
|  | 30032 | E001200 | Presenile dementia with paranoia |
|  | 37014 | Eu02200 | Dementia in Huntington's disease |
|  | 38286 | A411.00 | Jakob-Creutzfeldt disease |
|  | 108773 | F103z00 | Cerebral degeneration in disease NOS |
|  | 5651 | F11z.00 | Cerebral degeneration NOS |
|  | 24581 | F11x600 | Cerebral degeneration due to vitamin B12 deficiency |
|  | 99684 | F11x800 | Cerebral degeneration due to multifocal leucoencephalopathy |
|  | 18636 | E011200 | Wernicke-Korsakov syndrome |
|  | 62132 | E02y100 | Drug-induced dementia |
|  | 41185 | Eu02400 | Dementia in human immunodef virus [HIV] disease |
|  | 96549 | ZRV9.00 | Kendrick battery for detection of dementia in the elderly |
|  | 55023 | 66h..00 | Dementia monitoring |
|  | 1916 | E00..11 | Senile dementia |
|  | 55222 | ZS7C500 | Language disorder of dementia |
|  | 89036 | 9Ou3.00 | Dementia monitoring third letter |
|  | 57993 | Eu03.11 | Korsakov's psychosis, nonalcoholic |
|  | 47619 | Eu02z12 | Presenile psychosis NOS |
|  | 48091 | 2229.13 | O/E - senility - no psychosis |
|  | 49513 | E001100 | Presenile dementia with delirium |
|  | 52673 | A413.00 | Progressive multifocal leucoencephalopathy |
|  | 15165 | E001.00 | Presenile dementia |
|  | 105580 | C373D00 | Senile systemic amyloidosis |
|  | 48067 | TG44600 | Accident caused by ice pick |
|  | 7572 | F116.00 | Lewy body disease |
|  | 101999 | Eu84311 | Dementia infantalis |
|  | 54106 | Eu02100 | Dementia in Creutzfeldt-Jakob disease |
|  | 68194 | F21y211 | Binswanger's encephalopathy |
|  | 54505 | E012.00 | Other alcoholic dementia |
|  | 97422 | F11x.00 | Cerebral degeneration in other disease EC |
|  | 109834 | 8T05100 | Referral to dementia support organisation declined |
|  | 5095 | F21y200 | Binswanger's disease |
|  | 44341 | 9hD..00 | Exception reporting: dementia quality indicators |
|  | 70709 | 4L49.00 | Prion protein markers for Creutzfeldt-Jakob disease |
|  | 67762 | F103100 | Cerebral degeneration in mucopolysaccharidoses |
|  | 30641 | 9hD0.00 | Excepted from dementia quality indicators: Patient unsuitabl |
|  | 44592 | F11xz00 | Cerebral degeneration other disease NOS |
|  | 109737 | 8BM0200 | Dementia medication review |
|  | 64267 | Eu02y00 | Dementia in other specified diseases classif elsewhere |
|  | 48531 | F11x700 | Cerebral degeneration due to Jakob - Creutzfeldt disease |
|  | 59956 | F10z.00 | Childhood cerebral degenerations NOS |
|  | 26323 | Eu10711 | Alcoholic dementia NOS |
|  | 38438 | E001z00 | Presenile dementia NOS |
|  | 93372 | F103.00 | Cerebral degeneration in diseases EC |
|  | 29512 | F112.00 | Senile degeneration of brain |
|  | 109790 | 8CMe000 | Dementia advance care plan |
|  | 109708 | 8CMZ100 | Dementia care plan reviewed |
|  | 31892 | F11..00 | Other cerebral degenerations |
|  | 41089 | E002z00 | Senile dementia with depressive or paranoid features NOS |
|  | 53446 | Eu04100 | Delirium superimposed on dementia |
|  | 12621 | Eu02.00 | Dementia in other diseases classified elsewhere |
|  | 49674 | 9Ou1.00 | Dementia monitoring first letter |
|  | 1350 | E00..12 | Senile/presenile dementia |
|  | 4357 | Eu02z14 | Senile dementia NOS |
|  | 18386 | E002000 | Senile dementia with paranoia |
|  | 37015 | E003.00 | Senile dementia with delirium |
|  | 65235 | 9Ou5.00 | Dementia monitoring telephone invite |
|  | 47658 | F11x500 | Cerebral degeneration due to myxoedema |
|  | 109288 | A411000 | Sporadic Creutzfeldt-Jakob disease |
|  | 114501 | F10yz00 | Other cerebral degenerations in childhood NOS |
|  | 114978 | A410.00 | Kuru |
|  | 61405 | F426400 | Senile reticular degeneration |
|  | 110123 | 8CMZ300 | Dementia care plan review declined |
|  | 4500 | E011000 | Korsakov's alcoholic psychosis |
|  | 106311 | 8CMZ.00 | Dementia care plan |
|  | 34944 | Eu02z13 | Primary degenerative dementia NOS |
|  | 85853 | 9Ou..00 | Dementia monitoring administration |
|  | 42602 | E001000 | Uncomplicated presenile dementia |
|  | 39799 | Eu10600 | Mental and behave dis due to use alcohol: amnesic syndrome |
|  | 7323 | E000.00 | Uncomplicated senile dementia |
|  | 11106 | E011100 | Korsakov's alcoholic psychosis with peripheral neuritis |
|  | 92341 | R20z.00 | Senility, without psychosis NOS |
|  | 70957 | F11x400 | Cerebral degeneration due to neoplastic disease |
|  | 110075 | 8IAe200 | Dementia advance care plan review declined |
