# Supplementary material for: Associations between falls and other serious adverse events and antihypertensive medication in individuals with dementia: An observational cohort study
Source: PLoS Med. 2025 Sep 17;22(9):e1004731. doi: 10.1371/journal.pmed.1004731 (PMC12478963; doi:10.1371/journal.pmed.1004731)
Supplement: S4 Table — BP indicates blood pressure; CI, confidence interval; DBP, diastolic blood pressure; FI, frailty index; HDL, high-density lipoprotein; IMD, indices of multiple deprivation; SBP, systolic blood pressure. (DOCX) [file pmed.1004731.s005.docx]

| **Supplementary Table S4. Propensity score model in imputation datasets** | | | | | |
| --- | --- | --- | --- | --- | --- |
|  | | **With dementia** | | **Without dementia** | |
| **Variables** | | Odds ratio | 95% CI | Odds ratio | 95% CI |
| Patient characteristic | Age ≥65 years (vs. <65 years) | 0.75 | 0.65, 0.87 | 0.63 | 0.61, 0.64 |
|  | Gender (vs. male) | 1.46 | 1.25, 1.70 | 1.54 | 1.51, 1.58 |
| Ethnicity  (vs. white) | Black | 2.78 | 1.85, 4.20 | 2.79 | 2.17, 3.60 |
|  | South Asian | 1.25 | 0.79, 1.98 | 1.14 | 0.88, 1.46 |
|  | Other | 1.70 | 1.17, 2.46 | 1.40 | 1.07, 1.82 |
| Smoking status  (vs. non-smoker) | Ex-smoker | 1.18 | 1.07, 1.29 | 1.03 | 1.01, 1.06 |
|  | Current smoker | 0.89 | 0.78, 1.01 | 0.68 | 0.66, 0.70 |
| Alcohol consumption  (vs. non-drinker) | Trivial drinker (<1 unit/day) | 0.98 | 0.86, 1.12 | 0.90 | 0.87, 0.94 |
|  | Light drinker (1 to 2 units/day) | 1.08 | 0.94, 1.24 | 0.94 | 0.90, 0.98 |
|  | Moderate drinker (3 to 6 units/day) | 1.09 | 0.90, 1.33 | 0.97 | 0.93, 1.03 |
|  | Heavy drinker (≥7 units/day) | 0.84 | 0.47, 1.51 | 0.99 | 0.92, 1.07 |
|  | Not reported | 1.11 | 0.97, 1.27 | 1.02 | 0.98, 1.06 |
| IMD  (vs. IMD 1) | IMD 2 | 0.92 | 0.81, 1.05 | 1.02 | 1.00, 1.04 |
|  | IMD 3 | 0.91 | 0.81, 1.04 | 1.01 | 0.99, 1.03 |
|  | IMD 4 | 0.82 | 0.72, 0.94 | 1.03 | 1.00, 1.05 |
|  | IMD 5 | 0.90 | 0.77, 1.04 | 1.01 | 0.98, 1.04 |
| BMI  (vs. underweight of BMI <18.5) | Normal weight (BMI: 18.5 to 24.9) | 1.30 | 0.94, 1.80 | 1.13 | 0.80, 1.59 |
|  | Pre-obesity (BMI: 25.0 to 29.9) | 1.40 | 1.01, 1.95 | 1.30 | 0.96, 1.76 |
|  | Obesity (BMI ≥30) | 1.84 | 1.26, 2.69 | 1.59 | 1.22, 2.08 |
| SBP  (vs. <140 mmHg) | SBP (140 to 149 mmHg) | 1.22 | 1.08, 1.36 | 1.34 | 1.31, 1.36 |
|  | SBP (150 to 159 mmHg) | 1.84 | 1.57, 2.15 | 2.05 | 2.01, 2.10 |
|  | SBP (≥160 mmHg) | 2.88 | 2.55, 3.26 | 3.74 | 3.65, 3.82 |
| DBP  (vs. <80 mmHg) | DBP (80 to 89 mmHg) | 0.82 | 0.74, 0.91 | 0.98 | 0.96, 1.00 |
|  | DBP (≥90 mmHg) | 1.55 | 1.38, 1.74 | 2.22 | 2.17, 2.27 |
| Cholesterol | Total ≥6.2 mmol/L (vs. <6.2 mmol/L) | 0.82 | 0.70, 0.95 | 0.87 | 0.85, 0.89 |
|  | HDL ≥1.5 mmol/L (vs. <1.5 mmol/L) | 0.83 | 0.50, 1.39 | 1.02 | 0.94, 1.10 |
| Cardiovascular risk | QRisk2 score ≥10% (vs. <10%) | 3.47 | 2.57, 4.69 | 3.93 | 3.82, 4.04 |
| Frailty  (vs. Fit) | Mild frailty (FI: 0.120 to 0.239) | 1.35 | 1.03, 1.77 | 0.98 | 0.92, 1.05 |
|  | Moderate frailty (FI: 0.240 to 0.359) | 0.66 | 0.50, 0.88 | 0.69 | 0.64, 0.74 |
|  | Severe frailty (FI: ≥0.360) | 0.71 | 0.28, 1.77 | 0.46 | 0.36, 0.58 |
| Past medical history | Stroke | 1.28 | 1.08, 1.51 | 1.05 | 1.00, 1.09 |
|  | Myocardial infarction | 1.76 | 1.44, 2.15 | 2.36 | 2.26, 2.47 |
|  | Heart failure | 2.08 | 1.58, 2.74 | 2.18 | 2.65, 2.99 |
|  | Transient ischemic attack | 1.05 | 0.84, 1.32 | 0.97 | 0.92, 1.03 |
|  | Peripheral vascular disease | 1.16 | 0.86, 1.56 | 0.85 | 0.79, 0.91 |
|  | Angina | 1.90 | 1.63, 2.21 | 2.18 | 2.10, 2.26 |
|  | Coronary artery bypass graft | 1.34 | 0.91, 1.98 | 1.26 | 1.15, 1.39 |
|  | Chronic kidney disease | 2.77 | 2.22, 3.47 | 2.66 | 2.54, 2.78 |
|  | Diabetes mellitus | 1.44 | 1.27, 1.63 | 1.20 | 1.17, 1.23 |
|  | Atrial fibrillation | 1.61 | 1.37, 1.90 | 1.62 | 1.56, 1.68 |
|  | Cancer | 1.17 | 1.01, 1.36 | 1.07 | 1.03, 1.10 |
| Prescribed medications | Statins | 3.56 | 3.20, 3.95 | 3.52 | 3.46, 3.59 |
|  | Anti-thrombotics | 2.20 | 2.00 ,2.42 | 2.57 | 2.51, 2.62 |
|  | Anticholinergics | 0.87 | 0.77, 0.99 | 0.85 | 0.83, 0.87 |
|  | Antidepressants | 0.95 | 0.85, 1.06 | 1.14 | 1.12, 1.16 |
|  | Hypotonic/anxiolytics | 0.92 | 0.83, 1.03 | 0.97 | 0.95, 0.99 |
|  | Opioid | 0.82 | 0.75, 0.90 | 0.92 | 0.90, 0.93 |
| BP indicates blood pressure; CI, confidence interval; DBP, diastolic blood pressure; FI, frailty index; HDL, high density lipoprotein; IMD, indices of multiple deprivation; SBP, systolic blood pressure | | | | | |
