# Supplementary material for: Associations between falls and other serious adverse events and antihypertensive medication in individuals with dementia: An observational cohort study
Source: PLoS Med. 2025 Sep 17;22(9):e1004731. doi: 10.1371/journal.pmed.1004731 (PMC12478963; doi:10.1371/journal.pmed.1004731)
Supplement: S5 Table — BP indicates blood pressure; CI, confidence interval; DBP, diastolic blood pressure; FI, frailty index; HDL, high-density lipoprotein; IMD, indices of multiple deprivation; SBP, systolic blood pressure. (DOCX) [file pmed.1004731.s006.docx]

| **Supplementary Table S5. Propensity score model in the complete-case dataset** | | | | | |
| --- | --- | --- | --- | --- | --- |
|  | | **With dementia** | | **Without dementia** | |
| **Variables** | | Odds ratio | 95% CI | Odds ratio | 95% CI |
| Patient characteristic | Age ≥65 years (vs. <65 years) | 0.85 | 0.63, 1.14 | 0.87 | 0.84, 0.90 |
|  | Gender (vs. male) | 1.32 | 1.09, 1.59 | 1.43 | 1.38, 1.48 |
| Ethnicity  (vs. white) | Black | 2.48 | 1.20, 5.11 | 1.93 | 1.74, 2.14 |
|  | South Asian | 0.92 | 0.48, 1.74 | 1.18 | 1.08, 1.29 |
|  | Other | 1.83 | 0.94, 3.59 | 1.15 | 1.06, 1.26 |
| Smoking status  (vs. non-smoker) | Ex-smoker | 1.08 | 0.90, 1.29 | 0.96 | 0.93, 1.00 |
|  | Current smoker | 0.84 | 0.64, 1.09 | 0.73 | 0.69, 0.76 |
| Alcohol consumption  (vs. non-drinker) | Trivial drinker (<1 unit/day) | 0.91 | 0.73, 1.14 | 0.88 | 0.84, 0.92 |
|  | Light drinker (1 to 2 units/day) | 1.02 | 0.77, 1.33 | 0.96 | 0.91, 1.02 |
|  | Moderate drinker (3 to 6 units/day) | 0.93 | 0.65, 1.32 | 0.96 | 0.90, 1.02 |
|  | Heavy drinker (≥7 units/day) | 0.41 | 0.12, 1.37 | 1.05 | 0.93, 1.18 |
| Indices of multiple deprivation  (vs. IMD 1) | IMD 2 | 0.93 | 0.73, 1.14 | 1.02 | 0.97, 1.07 |
|  | IMD 3 | 0.87 | 0.68, 1.11 | 1.05 | 1.00, 1.11 |
|  | IMD 4 | 0.79 | 0.62, 1.02 | 1.05 | 1.00, 1.11 |
|  | IMD 5 | 1.04 | 0.79, 1.37 | 1.02 | 0.97, 1.08 |
| BMI  (vs. underweight of BMI <18.5) | Normal weight (BMI: 18.5 to 24.9) | 1.15 | 0.65, 2.04 | 1.25 | 1.08, 1.44 |
|  | Pre-obesity (BMI: 25.0 to 29.9) | 1.41 | 0.79, 2.51 | 1.58 | 1.37, 1.83 |
|  | Obesity (BMI ≥30) | 1.87 | 1.03, 3.39 | 2.11 | 1.83, 2.44 |
| SBP  (vs. <140 mmHg) | SBP (140 to 149 mmHg) | 1.40 | 1.13, 1.74 | 1.50 | 1.44, 1.56 |
|  | SBP (150 to 159 mmHg) | 2.33 | 1.81, 3.01 | 2.43 | 2.32, 2.55 |
|  | SBP (≥160 mmHg) | 4.43 | 3.43, 5.72 | 4.34 | 4.13, 4.56 |
| DBP  (vs. <80 mmHg) | DBP (80 to 89 mmHg) | 0.75 | 0.62, 0.91 | 0.92 | 0.89, 0.96 |
|  | DBP (≥90 mmHg) | 1.31 | 1.03, 1.67 | 1.84 | 1.75, 1.93 |
| Cholesterol | Total ≥6.2 mmol/L (vs. <6.2 mmol/L) | 0.76 | 0.62, 0.93 | 0.73 | 0.70, 0.76 |
|  | HDL ≥1.5 mmol/L (vs. <1.5 mmol/L) | 1.04 | 0.88, 1.24 | 1.01 | 0.98, 1.05 |
| Cardiovascular risk | QRisk2 score ≥10% (vs. <10%) | 2.27 | 1.24, 4.15 | 3.77 | 3.57, 3.97 |
| Frailty  (vs. Fit) | Mild frailty (FI: 0.120 to 0.239) | 1.24 | 0.72, 2.11 | 1.16 | 1.03, 1.32 |
|  | Moderate frailty (FI: 0.240 to 0.359) | 0.53 | 0.32, 0.88 | 0.75 | 0.67, 0.85 |
|  | Severe frailty (FI: ≥0.360) | 2.87 | 0.51, 16.24 | 0.47 | 0.30, 0.72 |
| Past medical history | Stroke | 1.37 | 1.00, 1.87 | 1.01 | 0.93, 1.09 |
|  | Myocardial infarction | 2.23 | 1.54, 3.22 | 2.36 | 2.17, 2.58 |
|  | Heart failure | 1.73 | 0.90, 3.32 | 3.79 | 3.24, 4.42 |
|  | Transient ischemic attack | 1.03 | 0.71, 1.50 | 0.90 | 0.81, 1.00 |
|  | Peripheral vascular disease | 1.00 | 0.62, 1.61 | 0.92 | 0.82, 1.04 |
|  | Angina | 2.02 | 1.53, 2.67 | 2.03 | 1.89, 2.18 |
|  | Coronary artery bypass graft | 1.70 | 0.90, 3.24 | 1.38 | 1.16, 1.62 |
|  | Chronic kidney disease | 2.81 | 1.98, 3.99 | 2.40 | 2.23, 2.59 |
|  | Diabetes mellitus | 1.20 | 0.97, 1.48 | 1.04 | 1.00, 1.08 |
|  | Atrial fibrillation | 1.65 | 1.16, 2.35 | 1.63 | 1.50, 1.78 |
|  | Cancer | 1.20 | 0.89, 1.63 | 1.01 | 0.95, 1.08 |
| Prescribed medications | Statins | 2.08 | 1.74, 2.48 | 2.01 | 1.94, 2.09 |
|  | Anti-thrombotics | 2.14 | 1.77, 2.59 | 2.28 | 2.20, 2.38 |
|  | Anticholinergics | 0.85 | 0.66, 1.11 | 0.76 | 0.72, 0.80 |
|  | Antidepressants | 0.81 | 0.65, 1.02 | 0.98 | 0.94, 1.02 |
|  | Hypotonic/anxiolytics | 0.76 | 0.60, 0.95 | 0.81 | 0.77, 0.84 |
|  | Opioid | 0.74 | 0.62, 0.89 | 0.82 | 0.80, 0.85 |
| BP indicates blood pressure; CI, confidence interval; DBP, diastolic blood pressure; FI, frailty index; HDL, high density lipoprotein; IMD, indices of multiple deprivation; SBP, systolic blood pressure | | | | | |
