# Supplementary material for: Associations between falls and other serious adverse events and antihypertensive medication in individuals with dementia: An observational cohort study
Source: PLoS Med. 2025 Sep 17;22(9):e1004731. doi: 10.1371/journal.pmed.1004731 (PMC12478963; doi:10.1371/journal.pmed.1004731)
Supplement: S6 Table — Data are mean ± SD or number (percentage). A standardised mean difference (SMD) of <0.1 suggests adequate variable balance after propensity score matching. *High deprivation indicates indices of multiple deprivation score of 5 (most deprived). †Thiazides includes thiazide-like diuretics. ‡Other antihypertensives includes centrally acting antihypertensives, direct renin inhibitors and vasodilators. ACE indicates angiotensin converting enzyme; ARBs, angiotensin Ⅱ receptor blockers; eFI, electronic frailty index; SMD, standardised mean difference. (DOCX) [file pmed.1004731.s007.docx]

| **Supplementary Table S6. Baseline characteristics of the study population before or after propensity score matching in the complete-case dataset, with standardised mean differences between exposure and control groups** | | | | | | | | | | | | | |
| --- | --- | --- | --- | --- | --- | --- | --- | --- | --- | --- | --- | --- | --- |
|  | With dementia | | | | | | Without dementia | | | | | | |
|  | Before matching | | | After matching | | | Before matching | | | After matching | | | |
|  | Antihypertensive prescription  (–) | Antihypertensive prescription  (+) | SMD | Antihypertensive prescription  (–) | Antihypertensive prescription  (+) | SMD | Antihypertensive prescription  (–) | Antihypertensive prescription  (+) | SMD | Antihypertensive prescription  (–) | Antihypertensive prescription  (+) | | SMD |
| Number | 2,261 | 1,195 |  | 973 | 973 |  | 76,313 | 30,897 |  | 25,441 | 25,411 | |  |
| **Characteristics** | | | | | | | | | | | | | |
| Age, yrs | 72.8±8.5 | 74.9±8.2 | 0.251 | 74.2±7.8 | 74.7±8.3 | 0.060 | 59.3±12.1 | 64.6±12.1 | 0.442 | 64.1±11.8 | | 64.1±12.2 | 0.002 |
| Female, n (%) | 1,207 (53.4) | 659 (55.1) | 0.035 | 544 (55.9) | 524 (53.9) | 0.041 | 34,763 (45.6) | 13,775 (44.6) | 0.019 | 11,570 (45.5) | | 11,385 (44.8) | 0.015 |
| Body mass index, kg/m^2^ | 25.8±4.7 | 26.7±5.0 | 0.193 | 26.2±4.9 | 26.4±4.8 | 0.052 | 27.8±5.7 | 29.0±5.9 | 0.207 | 28.5±5.8 | | 28.8±5.9 | 0.044 |
| Ethnicity |  |  | 0.103 |  |  | 0.041 |  |  | 0.052 |  | |  | 0.012 |
| White ethnicity, n (%) | 2,179 (96.4) | 1,133 (94.8) |  | 928 (95.4) | 928 (95.4) |  | 69,572 (91.2) | 27,927 (90.4) |  | 23,076 (90.7) | | 23,054 (90.6) |  |
| Black ethnicity, n (%) | 17 (0.8) | 22 (1.8) |  | 15 (1.5) | 13 (1.3) |  | 1,614 (2.1) | 843 (2.7) |  | 602 (2.4) | | 647 (2.5) |  |
| South Asian ethnicity, n (%) | 35 (1.5) | 19 (1.6) |  | 18 (1.9) | 16 (1.6) |  | 2,473 (3.2) | 1,160 (3.8) |  | 950 (3.7) | | 928 (3.6) |  |
| Other ethnicity, n (%) | 30 (1.3) | 21 (1.8) |  | 12 (1.2) | 16 (1.6) |  | 2,654 (3.5) | 967 (3.1) |  | 813 (3.2) | | 812 (3.2) |  |
| Indices of multiple deprivation |  |  | 0.050 |  |  | 0.047 |  |  | 0.049 |  | |  | 0.004 |
| Quintile 1, n (%) | 523 (23.1) | 285 (23.8) |  | 216 (22.2) | 221 (22.7) |  | 16,366 (21.4) | 6,026 (19.5) |  | 5,090 (20.0)) | | 5,055 (19.9) |  |
| Quintile 2, n (%) | 483 (21.4) | 259 (21.7) |  | 224 (23.0) | 211 (21.7) |  | 16,278 (21.3) | 6,627 (21.4) |  | 5,466 (21.5) | | 5,456 (21.4) |  |
| Quintile 3, n (%) | 489 (21.6) | 240 (20.1) |  | 188 (19.3) | 201 (20.7) |  | 15,575 (20.4) | 6,505 (21.1) |  | 5,255 (20.7) | | 5,277 (20.7) |  |
| Quintile 4, n (%) | 440 (19.5) | 225 (18.8) |  | 204 (21.0) | 196 (20.1) |  | 14,398 (18.9) | 6,062 (19.6) |  | 4,992 (19.6) | | 5,007 (19.7) |  |
| Quintile 5, n (%) | 326 (14.4) | 186 (15.6) |  | 141 (14.5) | 144 (12.1) |  | 13,696 (17.9) | 5,677 (18.4) |  | 4,638 (18.2) | | 4,646 (18.3) |  |
| Smoking status |  |  | 0.119 |  |  | 0.011 |  |  | 0.140 |  | |  | 0.010 |
| Non-smoker, n (%) | 1,074 (47.5) | 571 (47.8) |  | 458 (47.1) | 459 (47.2) |  | 32,543 (42.6) | 12,807 (41.5) |  | 10,515 (41.3) | | 10,566 (41.5) |  |
| Ex-smoker, n (%) | 858 (37.9) | 495 (41.4) |  | 400 (41.1) | 396 (40.7) |  | 25,506 (33.4) | 12,122 (39.2) |  | 9,614 (37.8) | | 9,668 (38.0) |  |
| Current smoking status, n (%) | 329 (14.6) | 129 (10.8) |  | 115 (11.8) | 118 (12.1) |  | 18,264 (23.9) | 5,968 (19.3) |  | 5,312 (20.9) | | 5,207 (20.5) |  |
| Alcohol consumption |  |  | 0.109 |  |  | 0.035 |  |  | 0.116 |  | |  | 0.012 |
| Nondrinker, n (%) | 539 (23.8) | 326 (27.3) |  | 252 (25.9) | 251 (25.8) |  | 16,583 (21.7) | 7,895 (25.6) |  | 6,252 (24.6) | | 6,310 (24.8) |  |
| Trivial, n (%) | 718 (31.8) | 349 (29.2) |  | 307 (31.6) | 296 (30.4) |  | 21,755 (28.5) | 7,947 (25.7) |  | 6,814 (26.8) | | 6,714 (26.4) |  |
| Light, n (%) | 343 (15.2) | 178 (14.9) |  | 146 (15.0) | 147 (15.1) |  | 11,042 (14.5) | 4,116 (13.3) |  | 3,475 (13.7) | | 3,467 (13.6) |  |
| Moderate, n (%) | 173 (7.7) | 84 (7.0) |  | 69 (7.1) | 73 (7.5) |  | 9,487 (12.4) | 3,447 (11.2) |  | 2,901 (11.4) | | 2,916 (11.5) |  |
| Heavy drinker, n (%) | 19 (0.8) | 4 (0.3) |  | 5 (0.5) | 4 (0.4) |  | 1,653 (2.2) | 561 (1.8) |  | 493 (1.9) | | 474 (1.9) |  |
| Not reported, n (%) | 469 (20.7) | 254 (21.3) |  | 194 (19.9) | 202 (20.8) |  | 15,793 (20.7) | 6,931 (22.4) |  | 5,506 (21.6) | | 5,560 (21.9) |  |
| QRisk2 score ≥10%, n (%) | 2,133 (94.3) | 1,178 (98.6) | 0.231 | 955 (98.2) | 956 (98.3) | 0.008 | 47,125 (61.8) | 27,925 (90.4) | 0.712 | 22,462 (88.3) | | 22,511 (88.5) | 0.006 |
| Frailty status |  |  | 0.133 |  |  | 0.022 |  |  | 0.142 |  | |  | 0.022 |
| Fit (eFI <0.12) | 2,154 (95.3) | 1,110 (92.9) |  | 919 (94.5) | 914 (93.9) |  | 74,541 (97.9) | 29,360 (95.0) |  | 24,506 (96.3) | | 24,403 (95.9) |  |
| Mildly frail (0.12≤ eFI < 0.24) | 38 (1.7) | 40 (3.3) |  | 21 (2.2) | 23 (2.4) |  | 786 (1.0) | 740 (2.4) |  | 428 (1.7) | | 487 (1.9) |  |
| Moderately frail (0.24≤ eFI < 0.36) | 67 (3.0) | 39 (3.3) |  | 31 (3.2) | 34 (3.5) |  | 918 (1.2) | 736 (2.4) |  | 468 (1.8) | | 511 (2.0) |  |
| Severely frail (0.36≤ eFI) | 2 (0.1) | 6 (0.5) |  | 2 (0.2) | 2 (0.2) |  | 68 (0.1) | 61 (0.2) |  | 39 (0.2) | | 40 (0.2) |  |
| Systolic blood pressure, mmHg | 144.1±11.7 | 150.2±13.4 | 0.484 | 148.9±12.7 | 149.3±13.3 | 0.034 | 141.6±10.9 | 149.8±13.5 | 0.668 | 147.7±12.7 | | 148.6±13.3 | 0.072 |
| Diastolic blood pressure, mmHg | 80.4±9.0 | 82.0±11.2 | 0.165 | 81.6±10.1 | 82.0±11.1 | 0.044 | 82.9±9.3 | 86.1±11.9 | 0.302 | 84.9±10.4 | | 85.8±11.7 | 0.075 |
| **Co-morbidities** | | | | | | | | | | | | | |
| Stroke, n (%) | 123 (5.4) | 126 (10.5) | 0.189 | 84 (8.6) | 92 (9.5) | 0.029 | 2,144 (2.8) | 1,705 (5.5) | 0.136 | 1,222 (4.8) | | 1,259 (4.9) | 0.007 |
| Transient ischemic attack, n (%) | 82 (3.6) | 76 (6.4) | 0.126 | 56 (5.8) | 62 (6.4) | 0.026 | 1,153 (1.5) | 941 (3.0) | 0.103 | 721 (2.8) | | 715 (2.8) | 0.001 |
| Myocardial infarction, n (%) | 61 (2.7) | 118 (9.9) | 0.299 | 54 (5.6) | 63 (6.5) | 0.039 | 1,051 (1.4) | 2,564 (8.3) | 0.327 | 944 (3.7) | | 1,365 (5.4) | 0.080 |
| Heart failure, n (%) | 20 (0.9) | 31 (2.6) | 0.131 | 16 (1.6) | 18 (1.9) | 0.016 | 282 (0.4) | 955 (3.1) | 0.210 | 255 (1.0) | | 458 (1.8) | 0.068 |
| Peripheral vascular disease, n (%) | 51 (2.3) | 47 (3.9) | 0.097 | 31 (3.2) | 34 (3.5) | 0.017 | 879 (1.2) | 753 (2.4) | 0.097 | 543 (2.1) | | 558 (2.2) | 0.004 |
| Coronary artery bypass graft, n (%) | 16 (0.7) | 46 (3.8) | 0.212 | 16 (1.6) | 24 (2.5) | 0.058 | 253 (0.3) | 606 (2.0) | 0.154 | 241 (0.9) | | 335 (1.3) | 0.035 |
| Angina, n (%) | 122 (5.4) | 200 (16.7) | 0.368 | 99 (10.2) | 117 (12.0) | 0.059 | 1,699 (2.2) | 3,189 (10.3) | 0.339 | 1,428 (5.6) | | 1,840 (7.2) | 0.066 |
| Atrial fibrillation, n (%) | 85 (3.8) | 99 (8.3) | 0.191 | 60 (6.2) | 63 (6.5) | 0.013 | 1,369 (1.8) | 1,943 (6.3) | 0.230 | 1,101 (4.3) | | 1,231 (4.8) | 0.024 |
| Diabetes mellitus, n (%) | 366 (16.2) | 285 (23.8) | 0.192 | 212 (21.8) | 211 (21.7) | 0.002 | 14,352 (18.8) | 9,633 (31.2) | 0.289 | 7,349 (28.9) | | 7,433 (29.2) | 0.007 |
| Chronic kidney disease, n (%) | 73 (3.2) | 120 (10.0) | 0.276 | 59 (6.1) | 74 (7.6) | 0.061 | 1,743 (2.3) | 2,471 (8.0) | 0.261 | 1,273 (5.0) | | 1,535 (6.0) | 0.045 |
| Cancer, n (%) | 147 (6.5) | 101 (8.5) | 0.074 | 85 (8.7) | 74 (7.6) | 0.041 | 3,819 (5.0) | 1,873 (6.1) | 0.046 | 1,509 (5.9) | | 1,540 (6.1) | 0.005 |
| **Treatment prescription** | | | | | | | | | | | | | |
| ACE inhibitors, n (%) | – | 536 (44.9) | – | – | 427 (43.9) | – | – | 15,613 (50.5) | – | – | | 12,348 (48.5) | – |
| ARBs, n (%) | – | 162 (13.6) | – | – | 132 (13.6) | – | – | 4,431 (14.3) | – | – | | 3,497 (13.7) | – |
| Calcium channel blockers, n (%) | – | 437 (36.6) | – | – | 339 (34.8) | – | – | 10,995 (35.6) | – | – | | 8,833 (34.7) | – |
| Thiazides, n (%)† | – | 444 (37.2) | – | – | 376 (38.6) | – | – | 9,299 (30.1) | – | – | | 7,846 (30.8) | – |
| Beta-blockers, n (%) | – | 433 (36.2) | – | – | 332 (34.1) | – | – | 10,559 (34.2) | – | – | | 8,225 (32.3) | – |
| Alpha-blocker, n (%) | – | 62 (5.2) | – | – | 46 (4.7) | – | – | 1,882 (6.1) | – | – | | 1,438 (5.7) | – |
| Other antihypertensives, n (%)‡ | – | 10 (0.8) | – | – | 7 (0.7) | – | – | 436 (1.4) | – | – | | 357 (1.4) | – |
| Statins, n (%) | 709 (31.4) | 687 (57.5) | 0.545 | 474 (48.7) | 499 (51.3) | 0.051 | 19,161 (25.1) | 16,519 (53.5) | 0.607 | 11,461 (45.0) | | 12,049 (47.4) | 0.046 |
| Antiplatelets/anticoagulants, n (%) | 710 (31.4) | 707 (59.2) | 0.581 | 481 (49.4) | 508 (52.2) | 0.056 | 12,796 (16.8) | 13,824 (44.7) | 0.636 | 8,871 (34.9) | | 9,566 (37.6) | 0.057 |
| Anticholinergics, n (%) | 328 (14.5) | 123 (10.3) | 0.128 | 109 (11.2) | 107 (11.0) | 0.007 | 10,157 (13.3) | 2,771 (9.0) | 0.138 | 2,554 (10.0) | | 2,462 (9.7) | 0.012 |
| Antidepressants, n (%) | 521 (23.0) | 209 (17.5) | 0.138 | 181 (18.6) | 179 (18.4) | 0.005 | 19,275 (25.3) | 5,991 (19.4) | 0.141 | 5,342 (21.0) | | 5,184 (20.4) | 0.015 |
| Hypotonic/anxiolytics, n (%) | 479 (21.2) | 180 (15.1) | 0.159 | 149 (15.3) | 155 (15.9) | 0.017 | 18,411 (24.1) | 5,172 (16.7) | 0.184 | 4,557 (17.9) | | 4,505 (17.7) | 0.005 |
| Opioids, n (%) | 782 (34.6) | 350 (29.3) | 0.114 | 292 (30.0) | 284 (29.2) | 0.018 | 30,354 (39.8) | 10,497 (34.0) | 0.120 | 8,958 (35.2) | | 8,855 (34.8) | 0.008 |
| Data are mean ± SD or number (percentage). A standardized mean difference (SMD) of <0.1 suggests adequate variable balance after propensity score matching. *High deprivation indicates indices of multiple deprivation score of 5 (most deprived). †Thiazides includes thiazide-like diuretics. ‡Other antihypertensives includes centrally acting antihypertensives, direct renin inhibitors, vasodilators, anti-anginal agent, endothelin receptor antagonist, phosphodiesterase type 5 inhibitor, prostacyclin analog and soluble guanylate cyclase stimulator. ACE indicates angiotensin converting enzyme; ARBs, angiotensin Ⅱ receptor blockers; eFI, electronic frailty index; SMD, standardized mean difference. | | | | | | | | | | | | | |
