# Supplementary material for: Associations between falls and other serious adverse events and antihypertensive medication in individuals with dementia: An observational cohort study
Source: PLoS Med. 2025 Sep 17;22(9):e1004731. doi: 10.1371/journal.pmed.1004731 (PMC12478963; doi:10.1371/journal.pmed.1004731)
Supplement: S7 Table — Exposure group and control group indicates with antihypertensive prescription and without antihypertensive prescription, respectively. CI indicates confidence interval; IPTW, inverse probability of treatment-weighted. (DOCX) [file pmed.1004731.s008.docx]

| **Supplementary Table S7. Hazard ratios of the initiation of antihypertensive medication drugs for each outcome in the complete-case dataset** | | | | | | | | | | |
| --- | --- | --- | --- | --- | --- | --- | --- | --- | --- | --- |
|  | **With dementia** | | | | | **Without dementia** | | | | |
|  | Exposure group | | Control group | |  | Exposure group | | Control group | |  |
|  | Population | Event | Population | Event | Hazard ratio (95%CI) | Population | Event | Population | Event | Hazard ratio (95%CI) |
| **Falls** (primary outcome) | | | | | | | | | | |
| Propensity score adjustment | 1,195 | 455 | 2,261 | 807 | 1.15 (1.01, 1.31) | 30,897 | 3,876 | 76,313 | 7,177 | 1.14 (1.09, 1.20) |
| Multivariable adjustment | 1,195 | 455 | 2,261 | 807 | 1.12 (0.98, 1.28) | 30,897 | 3,876 | 76,313 | 7,177 | 1.16 (1.11, 1.21) |
| Propensity score matching | 973 | 369 | 973 | 369 | 1.14 (0.99, 1.32) | 25,441 | 3,105 | 25,441 | 2,945 | 1.14 (1.08, 1.20) |
| IPTW | 1,195 | 455 | 2,261 | 807 | 1.14 (0.99, 1.32) | 30,897 | 3,876 | 76,313 | 7,177 | 1.17 (1.10, 1.25) |
| **Hypotension** | | | | | | | | | | |
| Propensity score adjustment | 1,195 | 79 | 2,261 | 103 | 1.22 (0.88, 1.70) | 30,897 | 778 | 76,313 | 932 | 1.46 (1.30, 1.63) |
| Multivariable adjustment | 1,195 | 79 | 2,261 | 103 | 1.21 (0.86, 1.70) | 30,897 | 778 | 76,313 | 932 | 1.47 (1.31, 1.64) |
| Propensity score matching | 973 | 61 | 973 | 54 | 1.20 (0.84, 1.74) | 25,441 | 579 | 25,441 | 437 | 1.40 (1.24, 1.59) |
| IPTW | 1,195 | 79 | 2,261 | 103 | 1.14 (0.81, 1.60) | 30,897 | 778 | 76,313 | 932 | 1.43 (1.25, 1.63) |
| **Syncope** | | | | | | | | | | |
| Propensity score adjustment | 1,195 | 42 | 2,261 | 84 | 0.91 (0.60, 1.37) | 30,897 | 564 | 76,313 | 1,175 | 1.17 (1.04, 1.31) |
| Multivariable adjustment | 1,195 | 42 | 2,261 | 84 | 0.88 (0.57, 1.35) | 30,897 | 564 | 76,313 | 1,175 | 1.19 (1.06, 1.33) |
| Propensity score matching | 973 | 30 | 973 | 42 | 0.80 (0.50, 1.28) | 25,441 | 460 | 25,441 | 406 | 1.22 (1.07, 1.40) |
| IPTW | 1,195 | 42 | 2,261 | 84 | 0.79 (0.52, 1.20) | 30,897 | 564 | 76,313 | 1,175 | 1.16 (0.99, 1.35) |
| **Fracture** | | | | | | | | | | |
| Propensity score adjustment | 1,195 | 217 | 2,261 | 384 | 1.20 (1.00, 1.45) | 30,897 | 2,590 | 76,313 | 6,745 | 1.03 (0.98, 1.08) |
| Multivariable adjustment | 1,195 | 217 | 2,261 | 384 | 1.17 (0.97, 1.41) | 30,897 | 2,590 | 76,313 | 6,745 | 1.02 (0.97, 1.08) |
| Propensity score matching | 973 | 183 | 973 | 169 | 1.20 (0.97, 1.47) | 25,441 | 2,150 | 25,441 | 2,215 | 1.02 (0.97, 1.09) |
| IPTW | 1,195 | 217 | 2,261 | 384 | 1.11 (0.91, 1.36) | 30,897 | 2,590 | 76,313 | 6,745 | 1.06 (0.99, 1.15) |
| Exposure group and control group indicates with antihypertensive prescription and without antihypertensive prescription, respectively. CI indicates confidence interval; IPTW, inverse probability treatment weighting. | | | | | | | | | | |
