# Supplementary material for: Associations between falls and other serious adverse events and antihypertensive medication in individuals with dementia: An observational cohort study
Source: PLoS Med. 2025 Sep 17;22(9):e1004731. doi: 10.1371/journal.pmed.1004731 (PMC12478963; doi:10.1371/journal.pmed.1004731)
Supplement: S8 Table — The results of Cox regression analyses adjusted for propensity score were shown. The number of individuals with dementia taking more than three antihypertensive medications were as follows: 3 drugs, n = 150; 4 drugs, n = 43; 5 drugs, n = 9; 6 drugs, n = 3; 7 drugs, n = 0. The number of individuals without dementia taking more than three antihypertensive medications were as follows: 3 drugs, n = 4,001; 4 drugs, n = 1,133; 5 drugs, n = 257; 6 drugs, n = 42; 7 drugs, n = 2. (DOCX) [file pmed.1004731.s009.docx]

| **Supplementary Table S8. Hazard ratios by the number of antihypertensive medications use for each outcome in the complete-case dataset** | | | | | | | | | | | | | | | | | | | |
| --- | --- | --- | --- | --- | --- | --- | --- | --- | --- | --- | --- | --- | --- | --- | --- | --- | --- | --- | --- |
|  | | **With dementia** | | | | | | | | **Without dementia** | | | | | | | | | |
| Number of  antihypertensive drugs | | Population | | Event | | Hazard ratio (95% CI) | | *P* value  for trend | | Population | | Event | | | Hazard ratio (95% CI) | | | *P* value  for trend | |
| **Falls** (primary outcome) | | | | | | | | | | | | | | | | | | | |
| 0 | 2,261 | | 807 | | – (reference) | | 0.140 | | | 76,313 | | | 7,177 | | | – (reference) | | | <0.001 |
| 1 | 581 | | 217 | | 1.09 (0.93, 1.27) | |  |  |  | 15,795 | | | 1,928 | | | 1.10 (1.04, 1.16) | | |  |
| 2 | 409 | | 161 | | 1.28 (1.07, 1.54) | |  |  |  | 9,667 | | | 1,217 | | | 1.14 (1.07, 1.22) | | |  |
| ≥3 | 205 | | 77 | | 1.15 (0.90, 1.47) | |  |  |  | 5,435 | | | 731 | | | 1.31 (1.20, 1.42) | | |  |
| **Hypotension** | | | | | | | | | | | | | | | | | | | |
| 0 | 2,261 | | 103 | | – (reference) | | 0.131 | | 76,313 | | 932 | | | – (reference) | | | <0.001 | | |
| 1 | 581 | | 35 | | 1.15 (0.77, 1.73) | |  |  | 15,795 | | 341 | | | 1.29 (1.13, 1.47) | | |  |  |  |
| 2 | 409 | | 28 | | 1.42 (0.90, 2.21) | |  |  | 9,667 | | 245 | | | 1.44 (1.23, 1.68) | | |  |  |  |
| ≥3 | 205 | | 16 | | 1.47 (0.84, 2.57) | |  |  | 5,435 | | 192 | | | 2.13 (1.80, 2.53) | | |  |  |  |
| **Syncope** | | | | | | | | | | | | | | | | | | | |
| 0 | 2,261 | | 84 | | – (reference) | | 0.688 | | | 76,313 | | | 1,175 | | | – (reference) | | | 0.006 |
| 1 | 581 | | 16 | | 0.71 (0.41, 1.24) | |  |  |  | 15,795 | | | 281 | | | 1.10 (0.96, 1.27) | | |  |
| 2 | 409 | | 18 | | 1.32 (0.77, 2.28) | |  |  |  | 9,667 | | | 182 | | | 1.23 (1.04, 1.46) | | |  |
| ≥3 | 205 | | 8 | | 0.96 (0.45, 2.06) | |  |  |  | 5,435 | | | 101 | | | 1.33 (1.07, 1.65) | | |  |
| **Fracture** | | | | | | | | | | | | | | | | | | | |
| 0 | 2,261 | | 384 | | – (reference) | | 0.508 | | | 76,313 | | | 6,745 | | | – (reference) | | | 0.136 |
| 1 | 581 | | 107 | | 1.14 (0.91, 1.42) | |  |  |  | 15,795 | | | 1,357 | | | 1.01 (0.95, 1.08) | | |  |
| 2 | 409 | | 74 | | 1.26 (0.97, 1.65) | |  |  |  | 9,667 | | | 799 | | | 1.03 (0.95, 1.11) | | |  |
| ≥3 | 205 | | 36 | | 1.10 (0.77, 1.57) | |  |  |  | 5,435 | | | 434 | | | 1.08 (0.98, 1.20) | | |  |
| The results of Cox regression analyses adjusted for propensity score were shown. The number of individuals with dementia taking more than three antihypertensive medications were as follows: 3 drugs, n=150; 4 drugs, n=43; 5 drugs, n=9; 6 drugs, n=3; 7 drugs, n=0. The number of individuals without dementia taking more than three antihypertensive medications were as follows: 3 drugs, n=4,001; 4 drugs, n=1,133; 5 drugs, n=257; 6 drugs, n=42; 7 drugs, n=2. | | | | | | | | | | | | | | | | | | | |
