# Supplementary material for: Associations between falls and other serious adverse events and antihypertensive medication in individuals with dementia: An observational cohort study
Source: PLoS Med. 2025 Sep 17;22(9):e1004731. doi: 10.1371/journal.pmed.1004731 (PMC12478963; doi:10.1371/journal.pmed.1004731)
Supplement: S9 Table — Exposure group and control group indicates with antihypertensive prescription and without antihypertensive prescription, respectively. CI indicates confidence interval; IPTW, inverse probability treatment weighting. (DOCX) [file pmed.1004731.s010.docx]

| **Supplementary Table S9. Hazard ratios of the initiation of antihypertensive medication drugs for a fall by the start date of the follow-up period.** | | | | | | | | | | |
| --- | --- | --- | --- | --- | --- | --- | --- | --- | --- | --- |
|  | **With dementia** | | | | | **Without dementia** | | | | |
|  | Exposure group | | Control group | |  | Exposure group | | Control group | |  |
|  | Population | Event | Population | Event | Hazard ratio (95%CI) | Population | Event | Population | Event | Hazard ratio (95%CI) |
| **Before 1st April 2014** | | | | | | | | | | |
| Propensity score adjustment | 3,974 | 1,509 | 19,232 | 6,728 | 1.15 (1.08, 1.22) | 130,671 | 14,325 | 953,716 | 71,813 | 1.08 (1.05, 1.10) |
| Multivariable adjustment | 3,974 | 1,509 | 19,232 | 6,728 | 1.11 (1.04, 1.19) | 130,671 | 14,325 | 953,716 | 71,813 | 1.09 (1.07, 1.12) |
| Propensity score matching | 3,721 | 1,426 | 3,721 | 1,324 | 1.13 (1.04, 1.24) | 120,213 | 13,066 | 120,213 | 13,332 | 1.05 (1.03, 1.08) |
| IPTW | 3,974 | 1,509 | 19,232 | 6,728 | 1.18 (1.09, 1.28) | 130,671 | 14,325 | 953,716 | 71,813 | 1.22 (1.18, 1.25) |
| **After 1st April 2014** | | | | | | | | | | |
| Propensity score adjustment | 88 | 24 | 216 | 53 | 1.15 (0.59, 2.27) | 11,714 | 359 | 100,121 | 1,288 | 1.25 (1.08, 1.46) |
| Multivariable adjustment | 88 | 24 | 216 | 53 | 1.00 (0.43, 2.30) | 11,714 | 359 | 100,121 | 1,288 | 1.25 (1.09, 1.44) |
| Propensity score matching | 57 | 15 | 57 | 13 | 1.08 (0.37, 3.13) | 10,602 | 310 | 10,602 | 273 | 1.19 (0.98, 1.44) |
| IPTW | 88 | 24 | 216 | 53 | 1.37 (0.67, 2.78) | 11,714 | 359 | 100,121 | 1,288 | 1.50 (1.21, 1.85) |
| Exposure group and control group indicates with antihypertensive prescription and without antihypertensive prescription, respectively. CI indicates confidence interval; IPTW, inverse probability treatment weighting. | | | | | | | | | | |
