# Supplementary material for: Associations between falls and other serious adverse events and antihypertensive medication in individuals with dementia: An observational cohort study
Source: PLoS Med. 2025 Sep 17;22(9):e1004731. doi: 10.1371/journal.pmed.1004731 (PMC12478963; doi:10.1371/journal.pmed.1004731)
Supplement: S1 Fig — Individuals were eligible for cohort entry if they met the following criteria: (1) aged ≥40 years old; (2) with qualifying first systolic BP levels of between 130 − 179 mmHg prior to the exposure period; (3) not having received any antihypertensives prior to the study start date; and (4) who were registered between 1st January 1998 and 31st December 2018 in CPRD GOLD. (DOCX) [file pmed.1004731.s011.docx]

**Supplementary Figure S1.** Definition of time periods used to define the cohort and follow-up periods

**
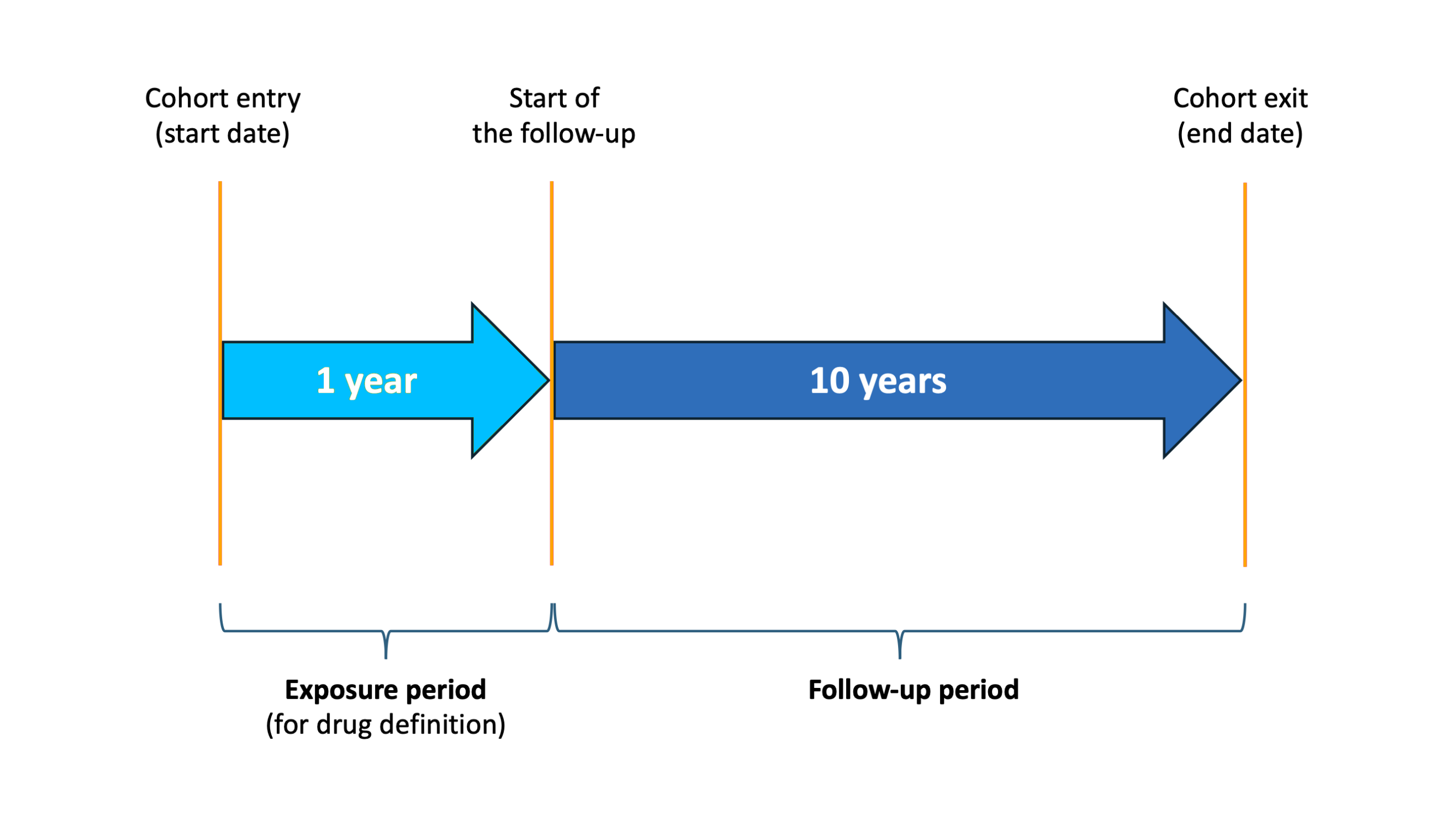
**
