# Supplementary material for: Associations between falls and other serious adverse events and antihypertensive medication in individuals with dementia: An observational cohort study
Source: PLoS Med. 2025 Sep 17;22(9):e1004731. doi: 10.1371/journal.pmed.1004731 (PMC12478963; doi:10.1371/journal.pmed.1004731)

**Supplementary Figure S2.** Flow diagram showing selection of patient records for inclusion in the study


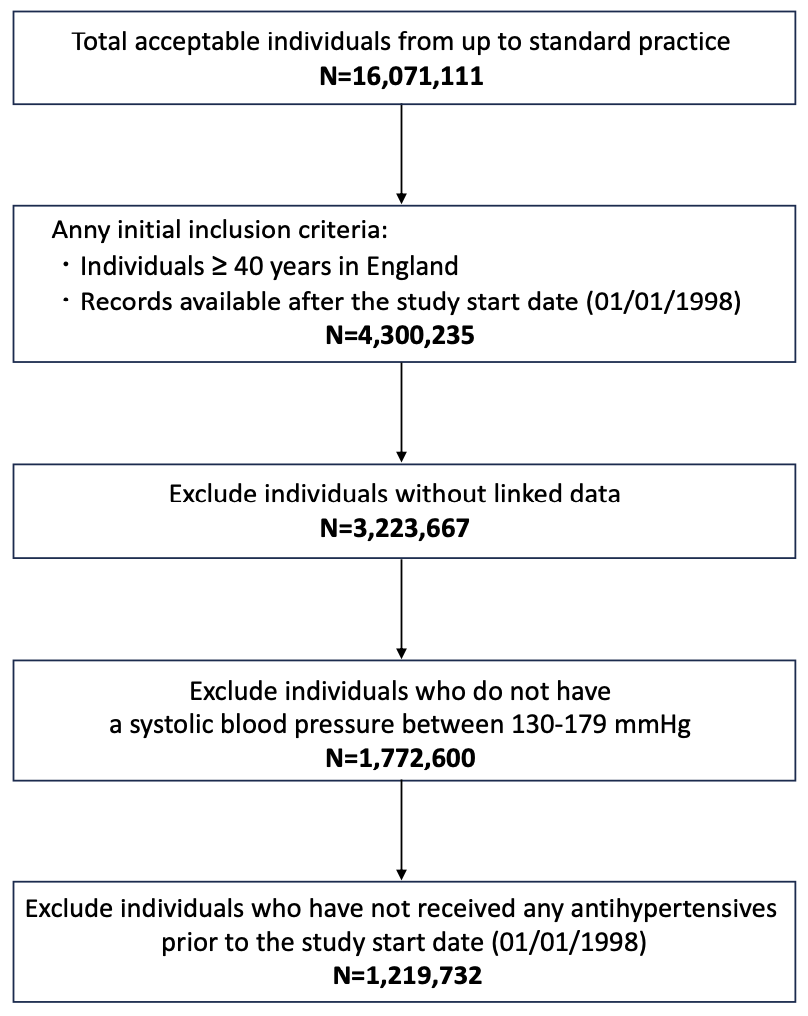

Supplement: S2 Fig — (DOCX) [file pmed.1004731.s012.docx]
