# Supplementary material for: Associations between falls and other serious adverse events and antihypertensive medication in individuals with dementia: An observational cohort study
Source: PLoS Med. 2025 Sep 17;22(9):e1004731. doi: 10.1371/journal.pmed.1004731 (PMC12478963; doi:10.1371/journal.pmed.1004731)
Supplement: S3 Fig — (A) the distribution of propensity scores among individuals with dementia, and (B) the distribution among those without dementia. (DOCX) [file pmed.1004731.s013.docx]

**Supplementary Figure S3.** Distribution of propensity scores by treatment status in individuals with and without dementia

**
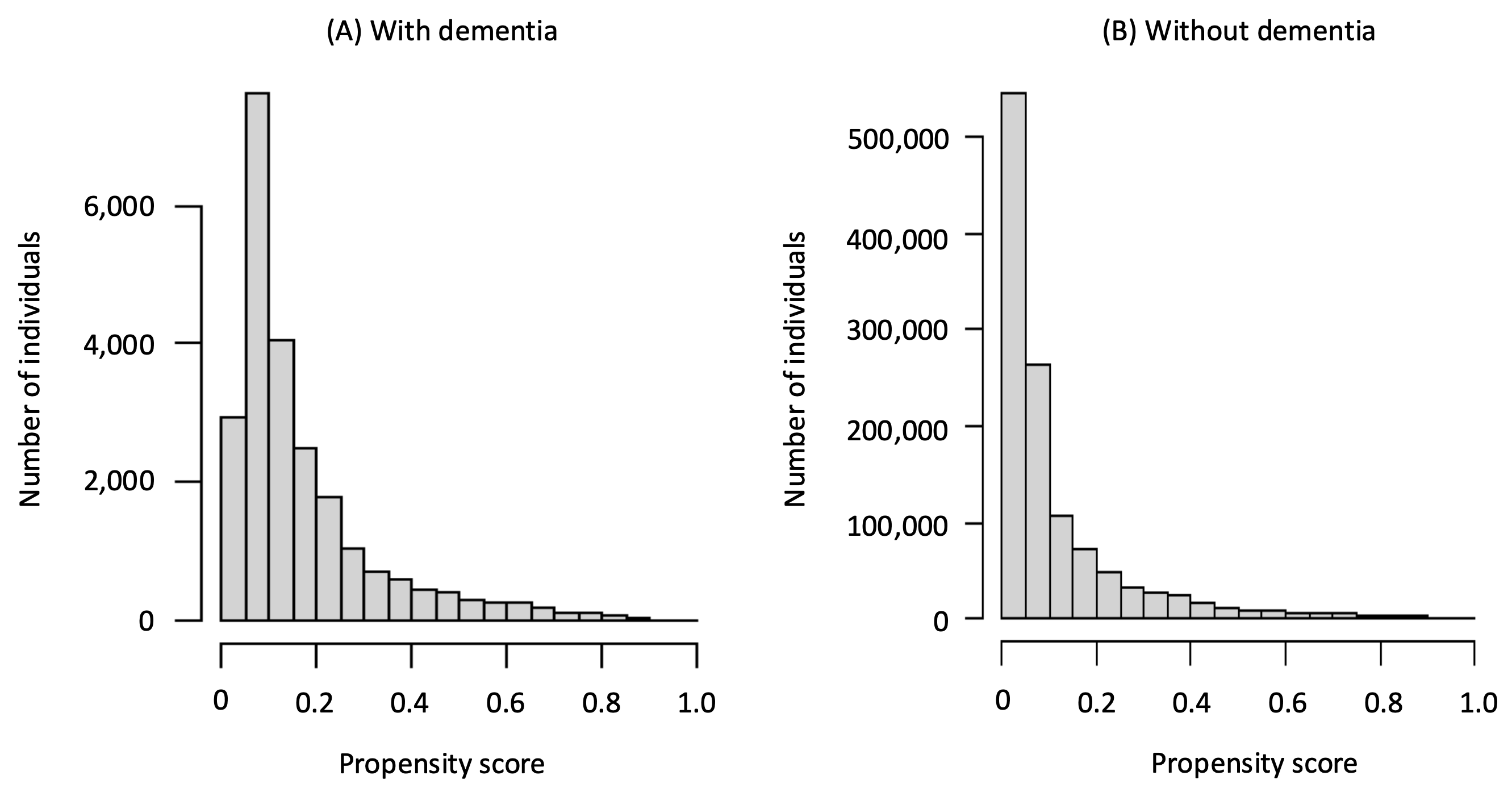
**
