# Supplementary material for: Associations between falls and other serious adverse events and antihypertensive medication in individuals with dementia: An observational cohort study
Source: PLoS Med. 2025 Sep 17;22(9):e1004731. doi: 10.1371/journal.pmed.1004731 (PMC12478963; doi:10.1371/journal.pmed.1004731)

**Supplementary Figure S4.** Cumulative risk of serious adverse events by antihypertensive medication according to categories of dementia and drug exposure status in the complete-case dataset


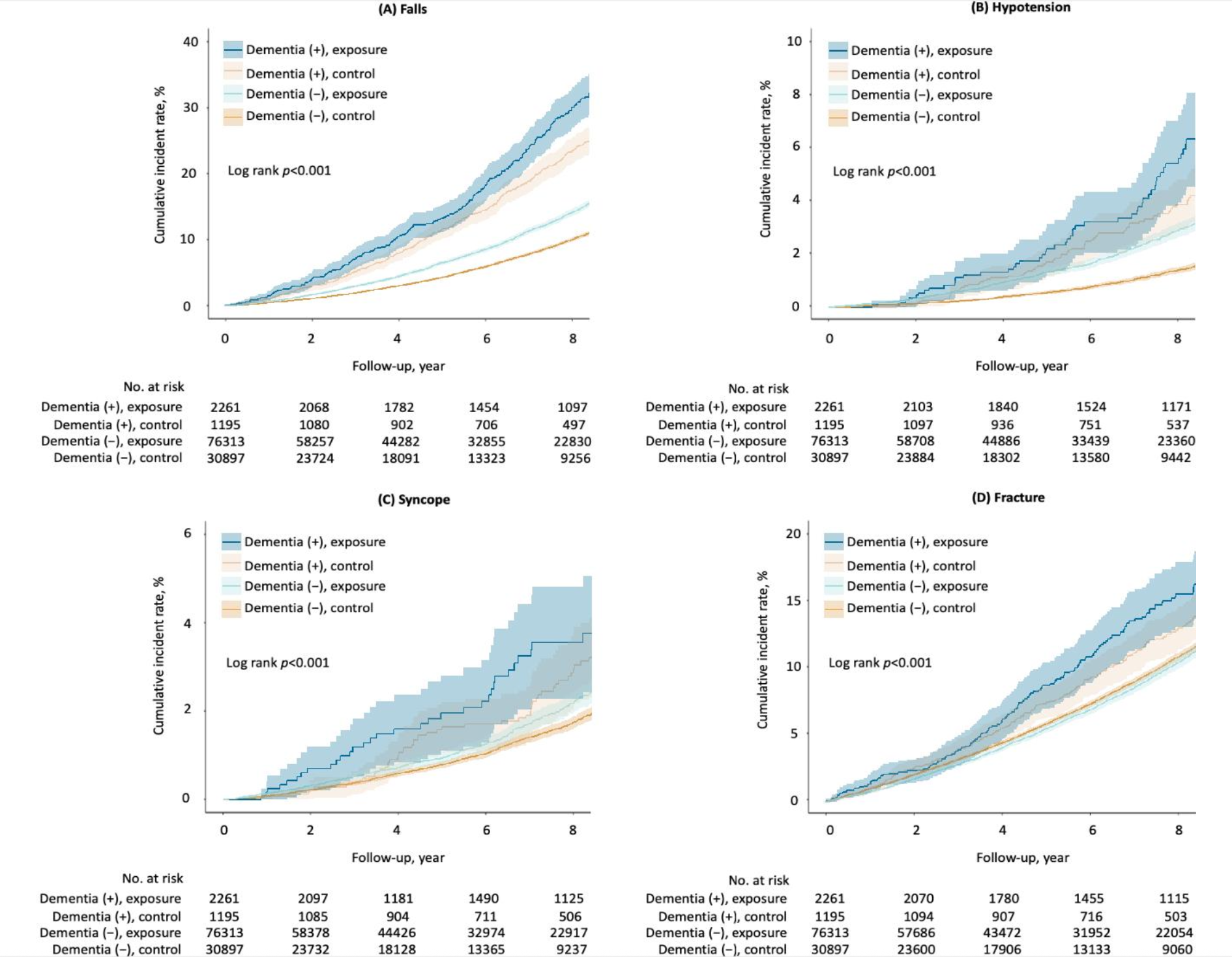

Supplement: S4 Fig — Kaplan–Meier curves of the cumulative incidence of serious adverse events by the four categorical groups in complete case are shown. S3A, S3B, S3C, and S3D Fig shows the cumulative incidence of falls, hypotension, syncope, and fracture, respectively. Each solid line indicates cumulative incident rate for each serious adverse event and its around area indicates the 95% confidence interval. (DOCX) [file pmed.1004731.s014.docx]
