# Supplementary material for: Associations between falls and other serious adverse events and antihypertensive medication in individuals with dementia: An observational cohort study
Source: PLoS Med. 2025 Sep 17;22(9):e1004731. doi: 10.1371/journal.pmed.1004731 (PMC12478963; doi:10.1371/journal.pmed.1004731)

**Supplementary Figure S5.** Differences in the risk of serious adverse events associated with antihypertensive treatment by dementia status in the complete-case dataset


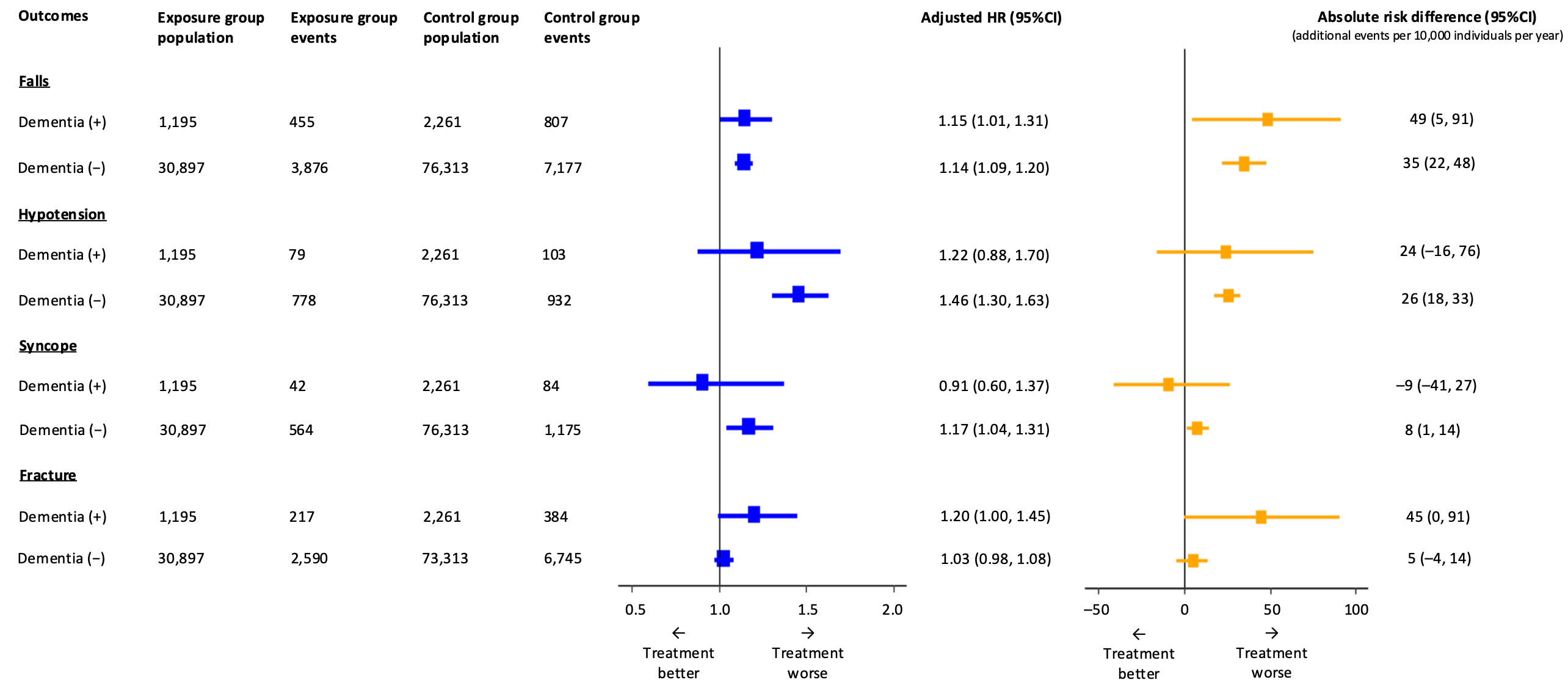

Supplement: S5 Fig — Models adjusted for propensity score using complete cases only. Absolute risk differences were described as additional events per 10,000 patients per year. CI indicates confidence interval; and HR, hazard ratio. (DOCX) [file pmed.1004731.s015.docx]
