# Supplementary material for: Associations between falls and other serious adverse events and antihypertensive medication in individuals with dementia: An observational cohort study
Source: PLoS Med. 2025 Sep 17;22(9):e1004731. doi: 10.1371/journal.pmed.1004731 (PMC12478963; doi:10.1371/journal.pmed.1004731)
Supplement: Attachment — Submitted filename: Response to reviewers_R.docx [file pmed.1004731.s018.docx]

**Ms. #PMEDICINE-D-25-00036R1: “Associations between falls and other serious adverse events and antihypertensive medication in individuals with dementia: An observational cohort study” by Fujiwara et al.**

**Responses to the Editor and Reviewers**

We thank the Editor and Reviewers for their thoughtful comments and advice, which helped us to substantially improve our manuscript. We have responded to the Editors’ and Reviewers’ comments below. New text in the manuscript is highlighted in yellow.

**The academic editor:**

***Q. The reviewers have raised some serious concerns that should be addressed. However, the issue of not examining 'benefits' is less concerning, as this is covered in the literature. Although this issue therefore does not need to be addressed in the analysis, they should include this in the discussion.***

**Response:**

We thank the Editor for the constructive comment regarding the discussion of potential benefits of antihypertensive treatment. In accordance with your comments, we have addressed this issue in the revised manuscript.

Specifically, in the Introduction, we have added a reference to recent evidence from a randomized controlled trial demonstrating that intensive blood pressure reduction was effective in lowering the risk of all-cause dementia among patients with hypertension, suggesting a possible preventive role for antihypertensive therapy.

Furthermore, we have revised the study limitations section to clarify that our study was not designed to evaluate the cardiovascular benefits of antihypertensive treatment in individuals with dementia. As such, we have noted that our findings should not be interpreted as a comprehensive risk-benefit assessment of antihypertensive therapy in this population.

**Page 10 Lines 130-133:**

Recent evidence from a randomized controlled trial demonstrated that intensive BP reduction was effective in lowering the risk of all-cause dementia among patients with hypertension, suggesting a potential preventive role for antihypertensive treatment.^16^

**Page 29 Lines 475-479:**

First, while antihypertensive treatment is known to reduce the risk of cardiovascular mortality, our study was not designed to evaluate the potential cardiovascular benefits of antihypertensive treatment in individuals with dementia. As such, our findings should not be interpreted as a comprehensive risk-benefit assessment of antihypertensive therapy in this population.

**Reviewer #1:**

***Q1. The data are what they are in an observational study, thank you for the revision.***

**Response:**

We thank the reviewer for their understanding and positive feedback regarding the limitations inherent to observational studies. We appreciate the reviewer's acknowledgment of our revisions.

**Reviewer #2:**

***Q1. Authors state that their primary aim is to examine the association between AHD and serious adverse events in individuals with dementia compared to those without dementia. Yet in statistical analysis section the association is assessed within each group, but no direct measure of comparison is indicated as outcome. Please improve data analysis on this point. In particular, regarding relative risk of adverse events, Authors conclude that it was "comparable between the two groups", but report among results, and in supplementary table S7, that significant interactions were observed in the association of primary outcome with antihypertensive treatment and dementia status. In Supplementary Table S7 actually it is reported that, in 3 out of the 4 parallel statistical methods they used, the interaction was significant. Therefore I understand that relative risk was actually greater, although slightly, among subjects with dementia. I feel that Table S7 should be reported as main result, and Authors should discuss why inverse probability weighting analysis does not confirm the increased relative risk in subjects with vs. without dementia.***

**Response:**

We appreciate the reviewer’s insightful comment regarding the clarity of our analytic strategy and interpretation of the interaction between antihypertensive treatment and dementia status.

First, we acknowledge that our primary aim was to compare the association of antihypertensive treatment with serious adverse events between individuals with and without dementia. As the reviewer correctly pointed out, in our main statistical models, we estimated the association of antihypertensive treatment with outcomes separately within each group. While this stratified approach allowed us to present group-specific estimates, it did not in itself constitute a formal statistical test of effect modification. To address this, we included interaction terms between dementia status and antihypertensive treatment in a set of supplementary models, the results of which are shown in Supplementary Table S7.

In this table, we report that the interaction term for the relative difference between dementia groups was statistically significant in 3 out of 4 modeling approaches, suggesting a slightly greater relative risk associated with antihypertensive treatment among individuals with dementia. However, the magnitude of the interaction effect was small. Given our large sample size, we agree that even modest relative differences may reach statistical significance, and thus, we interpreted these findings with caution. After careful deliberation, we opted not to emphasize Supplementary Table S7 as a main result in the manuscript for the following reasons:

1) Magnitude of Interaction: While statistically significant, the effect modification by dementia status was minimal in terms of relative effect size. We judged that this small difference may not be clinically meaningful and thus should not be the basis for our primary conclusion.
2) Inconsistency Across Models: One of the four analytical approaches—inverse probability of treatment weighting—did not yield a significant interaction. Given that inverse probability of treatment weighting is designed to reduce confounding by indication in observational settings in the same way as the other methods used, this inconsistency led us to interpret the interaction findings with caution.

Nonetheless, in response to the reviewer’s suggestion, we have now revised the Results sections to more clearly report the findings of Supplementary Table S7 and to explicitly state that, while statistical interaction was observed in some models, the clinical significance appears limited. We also now provide a more detailed rationale for the divergent findings across models, especially the non-significant result in the inverse probability of treatment weighting analysis.

**Page 21 Lines 335-337:**

While the interaction was statistically significant in three of the four analytical approaches, it was not significant in the inverse probability treatment weighting model (Supplementary Table S7).

***Q2. Regarding absolute risk increase, this appears to be clearly greater among dementia subjects, but no statistical test is reported to confirm this difference, which is presented as the main study result. Formal statistical testing should be reported for this primary outcome. Moreover, at lines 392-396 Authors discuss specific mechanisms explaining why "antihypertensive treatment could have adverse effects in individuals with dementia". Yet, according to the data, the incidence of the primary outcome was clearly greater in dementia subgroup irrespectively of AHD status (5x), and I feel that the Authors should state that this probably explains largely by himself the greater absolute risk observed in among subjects treated with AHD in the dementia vs. non-dementia subgroup (about 3.5x).***

**Response:**

We agree that the absolute risk increase associated with antihypertensive treatment appeared greater among individuals with dementia, and we have clarified this in the revised manuscript. While we did not conduct a formal statistical comparison of the absolute risk differences between the dementia and non-dementia subgroups due to the analytical complexity of pooling bootstrapped estimates across multiple imputed datasets, we have noted that the 95% confidence intervals for the absolute risk differences do not overlap, which provides indirect evidence of a meaningful difference.

We have explicitly added a statement that dementia itself may exacerbate vulnerability to treatment-related harms, as reflected in the greater absolute risk of falls observed among treated individuals with dementia compared to those without, despite similar relative risks.

**Page 25 Line 413 to Page 26 Line 417:**

Furthermore, our findings suggest that dementia itself may amplify the risk of serious adverse events associated with antihypertensive treatment. In our cohort, individuals with dementia had a markedly higher absolute risk of falls compared to those without dementia, despite similar relative risks, supporting the notion that dementia-related vulnerability may enhance the harms of treatment.

***Q3. Authors extensively discuss study limitations, but should better address the risk of unmeasured confounding, as the sample of subjects treated with AHD show a huge clinical difference, as expected, in comparison with the non-treated. In particular they are to be congratulated as they used parallel analyses, including propensity score adjusting and matching, which confirm that the association of AHD with hospitalization/death for fall, hypotension and syncope is independent of the greater clinical complexity of treated subjects. I feel that the use of propensity score should be cited in the Abstract too; they state that any potential effect of unmeasured confounding would be limited, as "the overall effects of antihypertensive treatment on serious adverse events in this population was similar to findings from meta-analyses of randomised controlled trials". Yet in this metaanalysis (that Authors should include among references: Albasri A, Hattle M, Koshiaris C, Dunnigan A, Paxton B, Fox S E et al. Association between antihypertensive treatment and adverse events: systematic review and meta-analysis BMJ 2021; 372: n189) the risk of falls was similar between treated and non-treated subjects. This suggests that at least part of the increased risk of death/hospital admission due to fall associate with AHD (irrespectively of dementia) in this sample is probably the expression of a "confounding by indication" linked to a greater vulnerability not fully captured by measured covariates; therefore, they should moderate their conclusions, suggesting that, due to observational design of the study, part of the increased risk might be explained by unmeasured confounding***

**Response:**

We thank the reviewer for their thoughtful comments and for acknowledging the strength of our parallel analyses using both propensity score adjustment and matching, which aimed to minimise confounding by clinical differences between treated and untreated individuals. We have added a brief mention of the results of the parallel analyses to the Abstract, as suggested.

**Page 5 Lines 64-70:**

In the primary analyses using propensity score adjustment, antihypertensive treatments were associated with a small increased risk of hospitalisation or death from falls (adjusted hazard ratio [aHR] 1.15, 95% confidence interval [CI] 1.08 to 1.22), hypotension (aHR 1.51, 95%CI 1.29 to 1.78), syncope (aHR 1.34, 95%CI 1.11 to 1.61), but not fracture (aHR 1.05, 95%CI 0.96 to 1.15), in individuals with dementia. These findings were consistent across different analytic approaches, including multivariable adjustment, propensity score matching, and inverse probability treatment weighting.

We fully agree that the risk of unmeasured confounding is an inherent limitation in observational studies and have already acknowledged this in the Discussion section. As noted by the reviewer, the treated population in our study displayed more clinical complexity at baseline—a reflection of treatment selection in real-world practice—which may introduce residual confounding despite extensive covariate adjustment. While we previously referenced findings from our prior study using the same dataset, which showed consistency with meta-analyses of randomized trials in older adults,^9^ we recognise the importance of also referencing the BMJ 2021 meta-analysis by Albasri A, et al., which reported no significant increase in fall risk associated with antihypertensive treatment. However, this meta-analysis included a smaller sample size and few studies with participants with dementia, possibly limiting the statistical power to detect an association. Falls are also less common in trial cohorts compared to the community-based cohort used in our study.

We have included this meta-analysis as an additional reference and revised the Discussion to more clearly acknowledge that part of the observed association may indeed reflect residual confounding, including confounding by indication.

However, after careful consideration, we believe that the primary conclusion of our study remains valid: that the absolute risk of serious adverse events associated with antihypertensive treatment is higher among individuals with dementia, even if part of this association may be influenced by unmeasured confounding.

**Page 32 Line 524-537:**

Seventh, although our propensity score analyses were successful in balancing the groups based on known confounding variables, we cannot rule out the presence of unmeasured confounding.^71,72^ While our previous work, which included the same individuals as the present study, demonstrated that the overall effects of antihypertensive treatment on serious adverse events in this population were similar to findings from meta-analyses of randomised controlled trials,^9^ other previous meta-analysis reported no significant association between antihypertensive treatment and fall risk.^72^ This discrepancy suggests that part of the increased risk of falls observed in our study, particularly in individuals with greater clinical complexity, could be due to residual confounding such as confounding by indication.^73^ It may also be explained by differences in the underlying study populations. Nevertheless, the relative comparison between individuals with and without dementia remains valid, since the treatment effects in both individuals with and without dementia would be subject to the same potential confounders.

***Q4. Authors state that they did not use a competing risks model to account for competing mortality risks, as according to similar analyses they performed in the same sample (ref 6), "this is unlikely to have resulted in substantially different results". This may well be the case. Yet, as one the main aims of AHD is to reduce vascular mortality, I feel that at least an adjusted comparison of total (and possibly vascular) mortality between treated and non-treated subjects should be provided to give the reader a clearer picture of vulnerability of the two sample and of possible trade-off between risk and benefits of AHD***

**Response:**

We thank the reviewer for this insightful comment. We agree that one of the main aims of antihypertensive treatment is to reduce cardiovascular mortality, and that comparing total and cardiovascular mortality between treated and untreated individuals would help clarify the trade-offs between benefits and risks. However, the evaluation of treatment benefits was beyond the scope of our current study and is well covered in the literature. In line with this guidance, we have now included an explicit statement in the Discussion section acknowledging that the present study was not designed to assess the cardiovascular benefits of antihypertensive therapy in individuals with dementia, and therefore cannot provide a complete evaluation of the risk-benefit balance.

**Page 29 Lines 475-479:**

First, while antihypertensive treatment is known to reduce the risk of cardiovascular mortality, our study was not designed to evaluate the potential cardiovascular benefits of antihypertensive treatment in individuals with dementia. As such, our findings should not be interpreted as a comprehensive risk-benefit assessment of antihypertensive therapy in this population.

***Q5. Authors should provide, possible in a supplementary table, separate figures of deaths and hospitalizations associated with different outcomes within the 2 subgroups, to give the reader a greater awareness of the events that are driving the observed associations and to better estimate trade-offs of treatment (I expect hospitalizations to have a greater weight than deaths)***

**Response:**

We thank the reviewer for this thoughtful suggestion. We agree that disaggregating hospitalisation and death could provide further insight into the nature of the serious adverse events and help readers better estimate the clinical trade-offs of antihypertensive treatment.

However, among those who experienced the primary outcome, only 0.44% of patients died without a preceding hospitalisation. Since the analyses are time to event, we only count the first event that occurs. For completeness we used a composite outcome but in practice this analysis primarily represents hospitalization. As a result, we do not think there is value in increasing the complexity of the results by adding disaggregated results by hospitalization or death.

***Q6. Please rephrase at line 71 and following:  "the absolute INCREASE OF FALL RISK ASSOCIATED with antihypertensive treatment per 10,000 individuals per year was significantly higher…".***

**Response:**

We have revised the abstract as your comments.

**Page 5 Lines 73-76:**

However, the absolute fall risk associated with antihypertensive treatment was significantly higher in individuals with dementia (47 per 10,000 individuals per year, 95%CI 26 to 70) compared to those without (14 per 10,000 individuals per year, 95%CI 10 to 18).

**Reviewer #3:**

***Q1. Were the analyses undertaken here all as specified in the study protocol? I particularly wanted to check that the estimate of absolute risk was pre-planned.*** 

**Response:**

As stated in the Methods section of the manuscript, this study was conducted under a pre-specified protocol approved by the Independent Scientific Advisory Committee (ISAC) for CPRD research (protocol number 19_042).

The estimation of absolute risks, including absolute risk differences and numbers needed to harm, were part of the planned analyses in the approved protocol. The models used to generate these risks were described in the protocol, reference to relative and absolute risks estimates was not explicitly made since it is standard good practice to generate both these measures in any analyses of treatment effects.

***Q2. How was death from other causes dealt with? Was this with censoring of the study period? Is this likely to represent a competing risk to the main outcomes in this study?*** 

**Response:**

We thank the reviewer for raising this important point. As described in the manuscript (Methods – Study population), individuals who died during the follow-up period were censored at the time of death unless death was the outcome of interest.

**Page 13 Lines 192-195:**

Individual’s characteristics were determined from information recorded at any point prior to the start of the follow-up period. Individuals exited the study on the study end date, when they transferred out of a registered CPRD practice, died, or experienced the specific outcome of interest (Supplementary Figure S1).

We acknowledge that death from causes other than the outcome of interest may act as a competing risk, particularly in older populations with multimorbidity. In a previous study using the same dataset,^6^ we performed analyses using the Fine-Gray competing risks model and showed that the results were highly consistent with those derived from traditional Cox models.

**Page 33 Lines 546-550:**

Tenth, we did not use a Fine-Gray competing risks model because our primary aim was to estimate cause-specific hazards rather than cumulative incidence, and individuals who died from unrelated causes were censored accordingly. Moreover, previous analyses using the same dataset have shown that results from Cox and Fine-Gray models were largely consistent,^9^ supporting the robustness of our approach.

***Q3. P12, L172. Was a fixed lookback period for BP used, or did this vary according to what data was available? To clarify, was the first hypertensive episode in each person's data used as the inception point/baseline for the study? How were people who stopped taking antihypertensives or changed dose dealt with?*** 

**Response:**

A fixed lookback period was in effect used to define baseline blood pressure. This was because blood pressure was part of the cohort inclusion criteria (i.e. a reading between 130-179 mm Hg) so for all patients, blood pressure was defined 12 months prior to the index date.

Regarding treatment changes, we used an intention-to-treat approach. Therefore, antihypertensive treatment status was defined at baseline, and we did not assess subsequent changes in treatment, including discontinuation or dose adjustments, during the follow-up period. We have clarified this as a limitation in the revised manuscript.

**Page 31 Lines 512-514:**

We also note that we did not evaluate subsequent treatment changes, including discontinuation or dose modification of antihypertensive drugs, during the follow-up period in the exposure group.

***Q4. P12, L175. Were these two exclusions made because these criteria indicated that these people had incomplete or poor-quality data?***

**Response:**

These exclusions were not made because the data were incomplete or of poor quality. Rather, they were applied to ensure the clinical appropriateness of the study population for evaluating the association between antihypertensive treatment and serious adverse events. Individuals without BP data could not be reliably assessed for treatment indication, and those with very high systolic BP would almost certainly require immediate antihypertensive therapy for ethical and clinical reasons. Including such individuals would have compromised the comparability between treated and untreated groups.

***Q5. P13, L183. Was dementia exposure defined just based on status at baseline? How were people who subsequently developed dementia dealt with in the analysis?***

**Response:**

As noted in our response to Q3 and in the manuscript, dementia status was defined at baseline, and the analysis followed an intention-to-treat approach. Therefore, individuals who developed dementia during the follow-up period were not reclassified, and new-onset dementia was not captured in our analysis. We have explicitly acknowledged this as a study limitation in the Discussion section.

**Page 30 Lines 490-493:**

Fourth, we used an “intention-to-treat approach” and did not account for individuals who developed new dementia during the observation period or who started antihypertensive treatment in the control group.

***Q6. P14, L213. How was the number of imputations decided? Was this in line with the estimates of fraction of missing information from the main analyses?*** 

**Response:**

As noted in the manuscript, we used 10 imputations for the multiple imputation analysis. Although the referenced guideline suggests that five imputations are often sufficient,^27^ we opted to perform 10 imputations to further reduce Monte Carlo error and enhance the stability of the pooled estimates across imputed datasets.

While we did not formally calculate the fraction of missing information prior to determining the number of imputations, we acknowledge that certain variables—such as ethnicity and cholesterol measures—had relatively high levels of missingness. Given this, and to ensure the robustness of our results, we selected 10 imputations in line with recommendation for settings involving moderate to high levels of missing data.^27^

***Q7. P15, L231. Why was a single-imputation strategy used for QRisk2, instead of including this in the MI model?***

**Response:**

In our dataset, QRisk2 scores were calculated for all individuals. In cases where the QRisk2 score was missing, it was replaced with the population mean of the QRisk2 score itself. This approach is consistent with how QRisk2 would be applied in routine clinical practice and was used only where necessary. It ensures that all individuals had a complete risk score, while maintaining consistency with real-world implementation and standard epidemiological practice.

***Q8. P16, L244. Was timing of start of baseline used in the matching process at all?
Was the propensity score estimated separately by dementia status?*** 

**Response:**

The timing of baseline was not explicitly included in the propensity score model or the matching process. Although we did not formally assess the distribution of baseline calendar year between treatment groups, given the large sample size and the population-based design of the dataset, we considered it unlikely that there were major differences in baseline timing that would introduce meaningful time-related confounding. Furthermore, our findings were similar in analyses regardless of whether matching was used or not, suggesting time-related confounding was not an issue.

Regarding the estimation of the propensity score, we have generated propensity scores separately within strata defined by dementia status. We accounted for potential differences in covariate structure and treatment assignment mechanisms between individuals with and without dementia.

***Q9. P17, L256. To clarify, was separate Cox model run for each level of dementia status, or just one model with a interaction between dementia status and antihypertensive exposure?***

**Response:**

We fitted separate Cox proportional hazards models within each dementia subgroup to estimate the association between antihypertensive treatment and each outcome. This stratified approach allowed us to obtain dementia-specific hazard ratios and absolute risk differences.

In addition, we conducted supplementary analyses including an interaction term between dementia status and antihypertensive treatment within a single model to formally test for effect modification. These results are presented in Supplementary Table S7.

To clarify this, we have added the following sentence to the original manuscript.

**Page 18 Lines 281-283:**

All four models were fitted separately for individuals with and without dementia to estimate dementia-specific associations between antihypertensive treatment and each outcome.

***Q10. P19, L302. Was there evidence of common support with respect to the propensity score?*** 

**Response:**

We thank the reviewer for this important question. While we did not present a formal visualisation of the propensity score distributions across treatment groups, we assessed the adequacy of common support using standardized mean differences (SMDs) for all covariates included in the propensity score model.

We have confirmed that all covariates achieved an SMD <0.1 after propensity score matching and weighting (see Tables 1 and S6), consistent with accepted thresholds for good balance. These results suggest that there was sufficient overlap in the distribution of propensity scores between treated and untreated individuals, and that comparisons were made among comparable populations.

***Q11. Table 3. How was the p-value for 'trend' generated? Is this an overall test of heterogeneity, or from including the number of drugs as a continuous variable?*** 

**Response:**

To evaluate the trend across increasing numbers of antihypertensive medications, we treated the categorical variable representing the number of antihypertensive drugs (0, 1, 2, ≥3) as an ordered factor in the Cox proportional hazards models. Specifically, we included the number of antihypertensive drugs as an ordered variable using the ordered() function in R, and fitted the model within each imputed dataset using the coxph() function. These models were then combined across imputed datasets using Rubin’s rules via the pool() function from the mice package. The reported p-value for trend reflects the significance of this ordered term in the pooled model, effectively testing for a linear association between the number of antihypertensive drugs and the hazard of each outcome.

**Reviewer #4:**

***Q1. Introduction
"However, caution should be exercised, as older people are at increased risk of serious adverse events, especially in those with progression of frailty"
To be fair, other evidence does suggest that individuals with frailty may also be at increased benefit from intensive blood pressure lowering,(1,2) and guidelines are not absolutely sure how to approach it but generally agree that hypertension treatment is still recommended.(3) Therefore, I think this statement is somewhat more debated than is currently suggested.
1.***      [***https://www.ahajournals.org/doi/10.1161/CIRCULATIONAHA.123.064003***](https://www.ahajournals.org/doi/10.1161/CIRCULATIONAHA.123.064003) ***2.***      [***https://www.ahajournals.org/doi/10.1161/HYPERTENSIONAHA.124.24214***](https://www.ahajournals.org/doi/10.1161/HYPERTENSIONAHA.124.24214) ***3.***      [***https://academic.oup.com/ageing/article/51/1/afab192/6410447?login=false***](https://academic.oup.com/ageing/article/51/1/afab192/6410447?login=false)

**Response:**

We thank the reviewer for this important comment and the helpful references. We agree that while older adults, especially those with advancing frailty, are at higher risk of serious adverse events, there is also emerging evidence suggesting that individuals with frailty may still derive benefit from antihypertensive treatment, including intensive BP lowering.

To reflect this more balanced perspective, we have revised the Introduction as follows.

**Page 9 Lines 115-120:**

Treating hypertension in older people is an important task for primary care physicians. Recent evidence suggests that individuals with frailty may still benefit from antihypertensive therapy,^6,7^ and a systematic review has shown that even frail older adults may derive cardiovascular benefit from BP lowering.^8^ However, caution should be exercised, as older people are at increased risk of serious adverse events, especially in those with progression of frailty.^9^

***Q2. Methods:
"Eligible individuals were those: (1) aged ≥40 years old; (2) with qualifying first systolic BP levels of between 130−179 mmHg9 prior to the exposure period; (3) not having received any antihypertensives prior to the study start date; and (4) who were registered between 1st January 1998 and 31st December 2018 in CPRD GOLD."
How can you be sure that individuals had not received antihypertensive medications prior to the study start date?***

**Response:**

We confirm that prior exposure to antihypertensive medications was assessed using the electronic health records available within the CPRD GOLD database, which contain longitudinal prescribing data from UK general practices. Individuals were excluded if there was any record of antihypertensive prescriptions at any point prior to the study entry date, based on retrospective review of their entire prescription history available in the database.

***Q3. Moreover, given the main study outcome of 'hospitalisation of death from a fall within 10 years of follow-up', what is the rationale for including individuals from age 40 or older? The event rates will be very low in the group <70, and since dementia generally occurs >65 years, this would create an artificial contrast when comparing the incidence rates in individuals with dementia and those without, which is mostly due to the age difference.***

**Response:**
We thank the reviewer for this insightful comment. We acknowledge that the incidence of serious adverse events is substantially lower among middle-aged individuals, and that dementia predominantly occurs in older populations. However, we chose to include individuals aged ≥40 years old to improve the generalisability of our findings and to reflect the full population of adults eligible for antihypertensive treatment in primary care.

CPRD GOLD is a nationally representative database of UK primary care patients, and limiting our sample to older adults only could have excluded clinically relevant populations, including those at risk of young-onset dementia and serious adverse events associated with antihypertensive treatment.

Importantly, any differences in age structure between the dementia and non-dementia groups were accounted for in our analyses, where we adjusted for age in all models and further controlled for baseline cardiovascular and frailty-related comorbidities.

***Q4. "Individuals characteristics were determined from information recorded at any point prior to the start of the follow-up period."
When did the 'follow-up' period start? Was the 'baseline' exposure period from 1998 to 1999? If individuals turned 40 after that, did they enter the database and then exit after 10 years? Because your mostly interested in events that generally occur in very old age, what would be the use of following someone from e.g. 1998 to 2008 from their 50th to their 60th, if they could also contribute to the analysis from their 60th to their 70th and be of much more interest to this research question?***

**Response:**

In our study, eligible individuals were those aged ≥40 years old who were registered in CPRD GOLD between 1 January 1998 and 31 December 2018. As illustrated in Supplementary Figure S1, each individual’s entry date was defined as the point at which they met all inclusion criteria and had at least one systolic BP measurement within the 130–179 mmHg range.

The exposure period lasted for one year from the individual entry date, during which antihypertensive treatment status was assessed. The follow-up period commenced immediately after the exposure period and continued for a maximum of 10 years. As such, both the exposure and follow-up periods varied across individuals depending on their entry date. Individuals who did not experience the outcome of interest were censored at the end of their 10-year follow-up.

We chose this design to construct a cohort that reflects a representative cross-section of the adult population across all age groups. By focusing on new prescriptions of antihypertensive treatment early after meeting eligibility criteria, this new user design enables a more robust evaluation of treatment-related risks and reduces the likelihood of confounding by indication. A fixed follow-up duration was then applied uniformly across individuals to ensure comparability in absolute risk estimation across exposure groups.

We have added the following to the revised manuscript to clarify the definitions of the exposure and follow-up periods in this study

**Page 13 Lines 181-188:**

The exposure and follow-up periods were defined relative to each individual's cohort entry date, with a one-year exposure assessment period followed by up to 10 years of follow-up. This design reflects a representative sample of the adult population across age groups and focuses on new users of antihypertensive treatment, allowing for a clearer assessment of treatment-related risks. A fixed follow-up duration was applied uniformly across individuals to ensure comparability of absolute risk estimates between exposure groups.

***Q5. "The exposure was prescription of any antihypertensive medication as defined in the British National Formulary (Supplementary Table S3). Individuals were allocated to the exposure group if they had been prescribed at least one antihypertensive medication during a 12-month period prior to the start date of the follow-up."
I think this is problematic for several reasons. 1.) The antihypertensive medications under study also include medications that are not primarily used for primary hypertension including loop diuretics and beta-blockers which are likely given (in combination with) heart failure, cardiac disease and/or arrythmia. This would cause confounding by indication in the analyses, because individuals with these types of conditions would have a worse prognosis a-priori. 2.) The propensity score matching/confounding adjustment might have captured some of this confounding by indication, but only in individuals whose diagnoses were accurately kept and dated in the database, so more information about the sensitivity of the database is essential. Furthermore, the propensity score adjusts for a combination of conditions, so these confounding by indication variables may be outweighed by other variables that are better predictors of the outcome but do not incur the same type of biases. It would be more prudent to exclude all individuals with relevant indications that may cause prescription of the included antihypertensive medication types of reasons other than hypertension altogether. 3.) Since antihypertensive treatment is generally given as long-term chronic treatment, considering individuals as exposed when having received 'at least one antihypertensive medication' during a 12-month period does not pertain to the individuals this study is interested in. Rather, those with single/sporadic treatment might have tried antihypertensive medication but did not tolerate it, felt that it was unnecessary, or received loop diuretics because of oedema. Furthermore, the 'unexposed' group may contain many individuals who received antihypertensive medication after the follow-up period. 

I wonder whether people with elevated blood pressure who do not receive subsequent antihypertensive treatment are comparable to those who do. The fact that they did not receive treatment may be itself an indication that the treating physician did not think they had hypertension (e.g. white coat hypertension), or that their blood pressure was normal a couple of visits later. Matching for blood pressure may not control for this type of indication bias, because the exact reason for refraining from antihypertensive treatment (as recommended by guidelines) is unknown.***

**Response:**

We thank the reviewer for this thorough and thoughtful critique. We address each point in turn below.

***Regarding the inclusion of medications such as beta-blockers and loop diuretics that may be prescribed for non-hypertensive indications:***

We agree with the reviewer that many antihypertensive medications, such as beta-blockers and loop diuretics, have multiple clinical indications beyond hypertension, including heart failure, arrhythmias, and angina. However, our aim was not to evaluate the efficacy or safety of antihypertensive treatment specifically for hypertension, but rather to examine the real-world association between the use of antihypertensive drugs and serious adverse events in clinical practice, regardless of indication.

Given the widespread overlap in indications for these medications, we believe that excluding individuals based on non-hypertensive indications would risk introducing selection bias and would limit the relevance of our findings to the very populations most vulnerable to treatment-related adverse outcomes. For instance, medications such as ACE inhibitors, ARBs, aldosterone antagonists, and calcium channel blockers are commonly used for both hypertension and heart failure. Beta-blockers, while less commonly used as first-line antihypertensives in recent years, were previously recommended as second-line agents in UK NICE guidance and were frequently prescribed for hypertension during our study period. Additionally, calcium channel blockers are widely used as both antihypertensives and anti-anginals, and alpha-blockers may be prescribed for both hypertension and benign prostatic hypertrophy.

There is no clear clinical boundary between conditions such as hypertensive heart disease, left ventricular hypertrophy, diastolic dysfunction, and heart failure with preserved ejection fraction—conditions that frequently coexist and evolve over time. Even excluding individuals with coded diagnoses of heart failure would not fully exclude those with these overlapping syndromes.

Importantly, all included medications would be expected to lower blood pressure irrespective of their indication, and it is this blood pressure lowering that may contribute to adverse outcomes such as falls. The risk of harm may be higher in individuals with comorbid conditions, and for that reason, we felt it important to include such individuals in our analyses. To account for potential confounding by indication, we adjusted for key comorbidities—such as heart failure, arrhythmias, and other cardiovascular conditions—in our propensity score models.

***Regarding the accuracy and sensitivity of diagnostic coding in the CPRD database:***

We acknowledge the potential limitations in diagnostic accuracy and date-stamping in electronic health record databases. However, CPRD GOLD is a high-quality, widely validated UK primary care database, and previous validation studies have shown acceptable levels of diagnostic accuracy for chronic conditions, including cardiovascular disease and dementia.^a,b,c^ Moreover, our analyses did not rely solely on diagnostic codes. We also incorporated prescription and measurement data, which are generally well recorded in CPRD. Notably, all issued prescriptions are automatically documented in the electronic health record, providing a reliable source of exposure information for phamacoepidemiological studies

***On defining exposure as having received at least one prescription during a 12-month period:***

We agree that this may include some individuals with only transient or trial use of antihypertensive medications. However, our exposure definition was designed to identify individuals initiating antihypertensive treatment in routine clinical practice, with the primary aim of assessing downstream risks following treatment initiation. Although treatment duration and adherence are clinically important considerations, they were not specifically evaluated in this study.

Nevertheless, in response to the reviewer’s comment, we have clarified in the revised manuscript that treatment persistence differed markedly between groups. Specifically, the median treatment duration was 6 years in the exposure group versus 0 years in the control group, with 28.9% of the control group initiating treatment during follow-up. This supports the interpretation that our comparison reflects greater versus lesser exposure to antihypertensive treatment, and that our effect estimates likely represent conservative estimates of the true treatment-associated risk.

We have added the following to the revised manuscript to clarify these points.

**Page 30 Lines 490-496:**

Fourth, we used an “intention-to-treat approach” and did not account for individuals who developed new dementia during the observation period or who initiated antihypertensive treatment in the control group (28.9% during follow-up). However, the median treatment duration was 6 years in the exposure group versus 0 years in the control group, suggesting that our effect estimates likely reflect conservative estimates of the true treatment-associated risk.

***Regarding potential biases in the untreated group (e.g., white-coat hypertension, spontaneous BP normalization):***

We agree that some individuals may not have been initiated on antihypertensive therapy for reasons unrelated to sustained elevated BP, which could reflect unmeasured clinical judgment or patient preferences. While this introduces the possibility of indication bias, our study was not restricted to individuals with confirmed or sustained hypertension. Rather, we sought to understand the potential harms associated with antihypertensive treatment use in the general adult population.

We believe that, although residual confounding is always possible in observational research, our multiple analytic strategies (propensity score adjustment, matching, and inverse probability treatment weighting) and extensive covariate adjustment provide robust support for the findings.

***Q6. Results:
"After matching, the SMD for all variables included in propensity score was reduced to below 0.1, indicating effective matching (Table 1 and 306 Supplementary Table S6)."
If you look at the individual (potentially) confounding characteristics, you see that many are significantly different, with the antihypertensive group consistently doing worse. Of course, these are small differences on a population level and only significant because of the large numbers but put together they may cause small differences in the outcome.***

**Response:**

We did not make any statistical comparisons of covariates in each group, for the reasons suggested by the reviewer (in large datasets even small differences achieve statistical significance). The values represented in Table 1 and Supplementary Table S6 and for the standardized mean differences (SMDs), not p values for difference between exposure and control group. SMDs are widely used to assess covariate balance in propensity score analyses, and an SMD <0.1 is generally considered to indicate negligible imbalance.^32^ In our matched cohort, all covariates included in the propensity score achieved very low SMDs <0.1 (Table 1 and Supplementary Table S6) which could be mistaken for p values, and these suggest that good balance was achieved across the measured variables. We have updated our tables to make the labelling more clear.

While we acknowledge that residual confounding cannot be entirely ruled out in observational studies, we believe that further balancing of covariates beyond this threshold would be unlikely to meaningfully improve the validity of the estimates.

**The titles of Table**

Table 1. Baseline characteristics of the study population before and after propensity score matching, with standardized mean differences between exposure and control groups

Supplementary Table S6. Baseline characteristics of the study population before or after propensity score matching in the complete-case dataset, with standardized mean differences between exposure and control groups

***Q7. Discussion
I think the elephant in the room here is that the outcomes only include those expected to be worse with antihypertensive treatment (death/hospitalisation from falls, hypotension, syncope), while they should be weighed against the potential gains (lower rates of mortality, cardiovascular disease, dementia etc.). With the information gathered by this study, it is impossible for clinicians to weigh the harms against the benefits. The absolute risk of falls may be higher but this may be compensated by greater longevity and/or fewer cardiovascular events. Randomized evidence does suggest that ceasing antihypertensive medication in cognitively frail older people results in poorer outcomes,(1) suggesting there is a benefit of taking these medications. Therefore, the current study does not sufficiently substantiate its conclusion that clinicians, patients and carers should consider the risks of antihypertensive treatment beyond what is already known.(2) It merely provides one side of the argument. What is really needed is a careful consideration of the harms versus the benefits.(2)
1.)***     [***https://pubmed.ncbi.nlm.nih.gov/38970547/***](https://pubmed.ncbi.nlm.nih.gov/38970547/) ***2.)***     [***https://pmc.ncbi.nlm.nih.gov/articles/PMC10978346/***](https://pmc.ncbi.nlm.nih.gov/articles/PMC10978346/)

**Response:**

We thank the reviewer for raising this important point regarding the need to consider both potential harms and benefits of antihypertensive treatment in individuals with dementia.

We fully agree that antihypertensive treatment has well-established benefits in reducing the risk of cardiovascular morbidity and mortality. However, our study was not designed to evaluate these potential cardiovascular benefits, particularly in the context of individuals with dementia. Instead, our primary aim was to investigate the potential risks—specifically, serious adverse events—associated with antihypertensive use in this population. Less common and adverse events are less well captured by clinical trials, and we felt an observational study could help with this side of the risk-benefit balance (essentially pharmacovigilance in this population). In contrast we felt our observational study was less well suited to identifying the intended cardiovascular effects of medications because of confounding by indication.^73^ We clearly state this as a limitation in the revised manuscript.

**Page 29 Lines 475-479:**

First, while antihypertensive treatment is known to reduce the risk of cardiovascular mortality, our study was not designed to evaluate the potential cardiovascular benefits of antihypertensive treatment in individuals with dementia. As such, our findings should not be interpreted as a comprehensive risk-benefit assessment of antihypertensive therapy in this population.

To acknowledge the need for a balanced interpretation, we have also incorporated additional text into the Discussion section to highlight relevant evidence supporting the potential benefits of antihypertensive treatment continuation in individuals with dementia.

**Page 27 Lines 434-438:**

Furthermore, a recent randomized controlled trial among long-term care residents with moderate to severe dementia suggested that discontinuation of antihypertensive treatment tended to increase the risk of serious adverse events, although this trial was not powered on clinical outcome events and had to be stopped early, precluding any definitive conclusions from being drawn.^47^

***Q8. "This is the first study to demonstrate that the absolute risk of serious adverse events with antihypertensive drugs differs by dementia status."
To warrant this conclusion, I think it would be far more appropriate to precisely match individuals with dementia to those without, and subsequently assess the risk of the primary outcome. Currently it is difficult to tell to what extent the differences between those with and without dementia are caused by the dementia group being very different form the other population in terms of age, comorbidity, and other relevant factors.***

**Response:**

We agree that directly comparing individuals with and without dementia requires careful consideration due to underlying differences in age, comorbidities, and clinical profiles. However, our primary aim was to estimate the effect of antihypertensive treatment on serious adverse events within each dementia subgroup rather than to make direct comparisons between individuals with and without dementia.

To this end, we performed propensity score matching and adjustment separately within each subgroup. This allowed us to appropriately balance measured confounders in treated and untreated individuals with dementia, and likewise within those without dementia. By using stratified analyses, we were able to obtain dementia-specific estimates of relative and absolute risk associated with antihypertensive treatment while avoiding the bias that could arise from forcing comparability between populations with fundamentally different baseline risks and clinical contexts.

We believe that this approach is the most appropriate for answering our research question, which focuses on how the harms of antihypertensive treatment may differ depending on dementia status.

***Q9. I think the discussion could do more to discuss the findings of antihypertensive trials in older cognitively frail populations (e.g. SPRINT, DANTON)(1,2) rather than narrowly focus on individuals with dementia, which is a very difficult group to include in hypertension RCTs.(3)
1.***      [***https://www.ahajournals.org/doi/10.1161/CIRCULATIONAHA.123.064003***](https://www.ahajournals.org/doi/10.1161/CIRCULATIONAHA.123.064003) ***2.***      [***https://pubmed.ncbi.nlm.nih.gov/38970547/***](https://pubmed.ncbi.nlm.nih.gov/38970547/) ***3.***      [***https://pmc.ncbi.nlm.nih.gov/articles/PMC10978346/***](https://pmc.ncbi.nlm.nih.gov/articles/PMC10978346/)

**Response:**

Our study specifically aimed to evaluate whether the risk of serious adverse events associated with antihypertensive treatment differs by dementia status. Dementia is a clinically distinct syndrome with unique cognitive, behavioral, and physiological implications that may modify vulnerability to treatment-related harms. Given this focus, our Discussion primarily addresses the risk-benefit profile of antihypertensive treatment specifically in individuals with dementia.

Nonetheless, we agree that findings from recent randomized trials in cognitively vulnerable populations, including those with frailty, offer important context for interpreting our results. In particular, the DANTON trial—which enrolled long-term care residents with moderate to severe dementia—found that discontinuation of antihypertensive treatment tended to increase the risk of serious adverse events. However, the DANTON trial was not powered to detect differences in serious adverse events and was terminated early, limiting the ability to draw definitive conclusions. The intervention also permitted systolic blood pressure to rise to levels (up to 180 mmHg) that may themselves carry risks. Therefore, we presented this trial as suggestive, but not conclusive, in the Discussion.

**Page 27 Lines 434-438:**

Furthermore, a recent randomized controlled trial among long-term care residents with moderate to severe dementia suggested that discontinuation of antihypertensive treatment tended to increase the risk of serious adverse events, although this trial was not powered on clinical outcome events and had to be stopped early, precluding any definitive conclusions from being drawn.^47^

***Q10. "In addition, this study did not include individuals with mild cognitive impairment who may also be at risk of serious adverse events."
This statement is not completely clear on whether these people excluded from the study or was mild cognitive impairment status simply not regarded in the analyses in any way.***

**Response:**

As noted in the Methods section (“Definitions of dementia”) and Supplementary Table S2, dementia status in this study was determined based on clinical codes recorded in the CPRD primary care database. Unfortunately, it is impossible to reliably identify individuals with mild cognitive impairment (MCI), as there were no consistently applied diagnostic codes for this condition in routine UK primary care records at that time.

Therefore, individuals with MCI were neither explicitly excluded nor specifically analyzed in this study. Rather, they were not identified as a distinct subgroup within the data and thus not included in our dementia-stratified analyses.

***Q11. "Third, we used an "intention-to-treat approach" and did not account for individuals who developed new dementia during the observation period or who started antihypertensive treatment in the control group."
I think this limitation merits a lot more discussion besides this simple sentence. Arguably, using a baseline exposure design with a 10-year follow-up is not the most appropriate approach for this research question. Why did you not use analysis with time varying covariates? This would avoid the problems with defining an exposure period, and because you would expect the relationship between antihypertensive medication use and falls to be more or less direct (as long as antihypertensives are taken, people are at increased risk), this would seem a more suitable approach at first glance. Explain why you chose the current method, and/or whether another approach like time-varying covariates would have been more appropriate.***

**Response:**

We appreciate the reviewer’s suggestion regarding the use of time-varying exposure models. While we agree that such models may provide additional insight under certain conditions, we opted for an intention-to-treat (ITT) design for the following reasons:

***Pragmatic constraints with large-scale real-world data:***

As with our prior research using the same CPRD cohort,^9^ the combination of time-varying covariates with propensity score adjustment and multiple imputation across over one million individuals poses methodological challenges. Incorporating time-varying exposure would require strong statistical assumptions about the timing, intensity, and reasons for treatment changes, which are not well characterized in the literature. These models also risk introducing additional biases that are difficult to quantify or correct for in real-world data.

***Conservativeness of effect estimates:***

Our analyses likely yield conservative estimates of risk, as individuals in the control group who initiated antihypertensive therapy during follow-up were not reclassified, and these late starters may have experienced similar risks. Indeed, our prior work has shown that the proportion of patients in the control group who initiate treatment during follow-up is relatively modest (28%), and treatment duration was substantially shorter compared with the baseline-treated group (median 0 years versus 6 years).^9^ Thus, the comparison of ever-treated vs. never-treated is likely to underestimate true treatment-related risks.

***Increasing confounding by indication:***

In real-world observational data such as CPRD, a key concern with time-varying exposure analysis is the risk of time-dependent confounding. Specifically, the decision to initiate antihypertensive treatment during follow-up may be influenced by a patient’s evolving clinical condition—such as rising blood pressure, new comorbidities, or increasing frailty—all of which may themselves be strong predictors of adverse outcomes. If such time-varying clinical factors are not fully captured or adjusted for in the model, the resulting estimates may be biased due to confounding by indication.

To clarify this rationale, we have expanded the Discussion section to include the following sentence.

**Page 30 Lines 490-505:**

Fourth, we used an “intention-to-treat approach” and did not account for individuals who developed new dementia during the observation period or who started antihypertensive treatment in the control group. More complex analyses using time-varying covariates could theoretically refine exposure classification^65^; however, integrating time-varying exposure into an analysis already involving multiple imputation and propensity score adjustment across a large, real-world dataset poses considerable methodological challenges, which are not well understood in the literature. Moreover, this approach likely yields conservative risk estimates, as control individuals who initiated treatment during follow-up were not reclassified, which may have attenuated observed treatment effects. In addition, time-varying exposure in observational data is highly susceptible to confounding by indication, as treatment initiation during follow-up may reflect worsening clinical status, thereby introducing bias that is difficult to fully address.

***Q12. "Ninth, we did not use a Fine-Gray competing risks model to account for competing risks, although previous analyses using the same data suggest this is unlikely to have resulted in substantially different results."
Rather than mentioning this as a limitation, please explain why not.***

**Response:**

Fine-Gray subdistribution hazard models are most useful for estimating cumulative incidence in the presence of competing risks; however, they yield different interpretations and are not always the preferred method when the goal is to examine etiologic associations rather than prediction. In our study, individuals who died from unrelated causes were censored, consistent with a cause-specific hazard approach.

Additionally, as noted in the manuscript, we have previously conducted similar analyses using the same CPRD dataset, incorporating both Cox and Fine-Gray models,^9^ and found that the main results were largely consistent across both methods. This provides further reassurance that our choice of modeling approach does not materially affect the conclusions of the study.

We have revised the manuscript to better clarify this rationale in place of framing it solely as a limitation.

**Page 33 Lines 546-550:**

Tenth, we did not use a Fine-Gray competing risks model because our primary aim was to estimate cause-specific hazards rather than cumulative incidence, and individuals who died from unrelated causes were censored accordingly. Moreover, previous analyses using the same dataset have shown that results from Cox and Fine-Gray models were largely consistent,^9^ supporting the robustness of our approach.

***Q13. "Tenth, the absence of individual BP values in our dataset meant that we could not assess potential relationships between increased BP variability and the risk of falls."
If blood pressure values were missing from the dataset, how did you assess the systolic blood pressure levels between 130-179?***

**Response:**

The blood pressure value used for inclusion (systolic blood pressure 130–179 mmHg) was defined as the first qualifying blood pressure measurement recorded in the electronic health record. This could represent a single measurement, or in some cases, an average of multiple readings taken at a clinic visit or at home, depending on how it was entered by the clinician. However, the specific structure or source of each BP value (e.g., whether it reflected multiple readings) could not be reliably determined from the data.

After careful consideration, we recognize that our study was not designed to assess blood pressure variability, and that we do not have sufficient information to quantify or meaningfully interpret blood pressure variability across individuals. Accordingly, we have removed the original Limitation paragraph that referred to the absence of individual blood pressure values (previously listed as “Eleventh”) from the revised manuscript.

***Q14. Would it have been interesting to assess whether the results differed in subgroups according to blood pressure preceding treatment initiation? (e.g. <150,150-160,>160 mmHg)***

**Response:**

We appreciate the reviewer’s thoughtful suggestion. We agree that subgroup analyses stratified by baseline systolic blood pressure levels (e.g., <150, 150–160, >160 mmHg) may provide additional insights into potential effect modification. However, our primary aim was to examine the overall association between antihypertensive treatment and serious adverse events in individuals with and without dementia, rather than to evaluate treatment effects across different BP thresholds.

Furthermore, our dementia subgroup included approximately 23,000 individuals, of whom only around 4,000 were exposed to antihypertensive treatment. Further stratifying this already limited sample would have substantially reduced statistical power, limiting our ability to detect meaningful differences across BP categories. For these reasons, we did not perform blood pressure-stratified analyses in the current study.

**The statistical reviewer:**

***Q1. If the authors have a comparison of the difference between dementia/non-dementia of the association between AHD and SAEs they should do this directly. The most powerful way to accomplish this would be to include an interaction between AHD and dementia status, and test for evidence of difference in the beta coefficient.***

**Response:**

We thank the statistical reviewer for this important point regarding the most rigorous approach to evaluating whether the association between antihypertensive drug use and serious adverse events differs by dementia status.

We have received a similar comment from Reviewer #2, but we had already included such interaction terms in supplementary models, the results of which are presented in Supplementary Table S7. These models were constructed to assess whether dementia status modified the association between antihypertensive treatment and serious adverse events across our four analytic approaches. As reported, the interaction term was statistically significant in three of the four methods, including the multivariable-adjusted, propensity score-adjusted, and propensity score–matched models. However, the interaction term was not statistically significant in the inverse probability of treatment weighting model, which we regard as an important inconsistency given inverse probability of treatment weighting’s ability to balance observed covariates across groups.

We acknowledge that the β coefficient for the interaction term represents the estimated difference in the log hazard ratio between individuals with and without dementia. While the direction of the effect suggests a slightly higher risk among individuals with dementia, we judged that this difference, although statistically significant in some models—likely due to the large sample size—may not be clinically meaningful. Therefore, we chose not to highlight the interaction analysis as a primary result in the main text.

***Q2. The time-varying exposure is a good point – particularly given that many people will commence treatment with an AHD as they age. The gold-standard for dealing with this is the application of ‘G methods’. Using a fixed exposure covariate is a problem where someone’s outcome at an earlier time-point affects their exposure at a later time-point, e.g. someone experiences a fall and their AHD is changed to prevent hypotension and dizziness. These are fairly complicated methods – it would be ideal to use one of the ‘g methods’ to deal with AHD as a time-varying exposure, but at the very least the potential bias caused from a fixed exposure should be acknowledged as a limitation of study design.***

**Response:**

We appreciate the reviewer’s thoughtful comment highlighting the issue of exposure misclassification and structural confounding that can arise when antihypertensive drug exposure varies over time. We agree that this is an important methodological consideration, particularly in real-world data where treatment status may change in response to clinical events such as falls, hypotension, or other adverse outcomes.

In line with this suggestion, we have expanded the Discussion to acknowledge the potential for structural confounding when using fixed exposure models.

**Page 30 Lines 490 to Page 31 Line 510:**

Fourth, we used an “intention-to-treat approach” and did not account for individuals who developed new dementia during the observation period or who started antihypertensive treatment in the control group. More complex analyses using time-varying covariates could theoretically refine exposure classification^65^; however, integrating time-varying exposure into an analysis already involving multiple imputation and propensity score adjustment across a large, real-world dataset poses considerable methodological challenges, which are not well understood in the literature. Moreover, this approach likely yields conservative risk estimates, as control individuals who initiated treatment during follow-up were not reclassified, which may have attenuated observed treatment effects. In addition, time-varying exposure in observational data is highly susceptible to confounding by indication, as treatment initiation during follow-up may reflect worsening clinical status, thereby introducing bias that is difficult to fully address. In addition, when exposure status changes over time and may itself be influenced by prior outcomes, fixed-exposure models may be particularly vulnerable to structural confounding. While causal methods such as G-methods have been developed to address this issue,^66^ their implementation would require further assumptions and complexity that go beyond the scope of the current study.

***Q3. I think the manuscript does a reasonable job of talking about the limits from unmeasured confounding in the discussion in terms of a fixed exposure. If the next version doesn’t include time-varying covariate with appropriate methods they should also acknowledge the issue of confounding that can occur when the exposure varies over time but is treated as fixed in the analysis.***

**Response:**

As described in our response to Reviewer #3 (Q11), we used an intention-to-treat design based on baseline exposure status, following individuals for up to 10 years. While we acknowledge that time-varying exposure models could, in theory, offer more dynamic classifications of treatment status, incorporating such methods into a framework already employing multiple imputation and propensity score adjustment across a large, real-world dataset would introduce substantial methodological complexity and require strong assumptions about treatment timing and indications—assumptions that are difficult to justify in this setting.

Nevertheless, in response to the reviewer’s suggestion, we have revised the manuscript to better acknowledge this limitation. Specifically, we now explicitly state that by treating exposure as fixed over time, our analysis may be vulnerable to additional unmeasured confounding arising from treatment changes during follow-up that are not captured in our model.

**Page 31 Lines 510-512:**

Furthermore, by treating exposure as fixed over time, our analysis may be subject to additional unmeasured confounding arising from changes in treatment status that are not captured in our model.

**Reference**

6. Wang Z, Du X, Hua C, Li W, Zhang H, Liu X, Wang Y, Jiang C, Guo J, Lv Q, Anderson CS, Dong J, Ma C. The Effect of Frailty on the Efficacy and Safety of Intensive Blood Pressure Control: A Post Hoc Analysis of the SPRINT Trial. *Circulation.* 2023;148:565-574.

7. Chen L, You S, Ee N, Rockwood K, Ward DD, Woodward M, Liu T, Gao Y, Williamson JD, Anderson CS, Harris K, Chen X, Peters R. Impact of Frailty on Antihypertensive Treatment in Older Adults. *Hypertension.* 2025;82:509-519.

8. Bogaerts JMK, von Ballmoos LM, Achterberg WP, Gussekloo J, Streit S, van der Ploeg MA, Drewes YM, Poortvliet RKE. Do we AGREE on the targets of antihypertensive drug treatment in older adults: a systematic review of guidelines on primary prevention of cardiovascular diseases. *Age Ageing.* 2022;51:afab192.

9. Sheppard JP, Koshiaris C, Stevens R, Lay-Flurrie S, Banerjee A, Bellows BK, Clegg A, Hobbs FDR, Payne RA, Swain S, Usher-Smith JA, McManus RJ. The association between antihypertensive treatment and serious adverse events by age and frailty: A cohort study. *PLoS Med.* 2023;20:e1004223.

16. He J, Zhao C, Zhong S, Ouyang N, Sun G, Qiao L, Yang R, Zhao C, Liu H, Teng W, Liu X, Wang C, Liu S, Chen CS, Williamson JD, Sun Y. Blood pressure reduction and all-cause dementia in people with uncontrolled hypertension: an open-label, blinded-endpoint, cluster-randomized trial. *Nat Med.* 2025; Online ahead of print.

27. Sterne JA, White IR, Carlin JB, Spratt M, Royston P, Kenward MG, Wood AM, Carpenter JR. Multiple imputation for missing data in epidemiological and clinical research: potential and pitfalls. *BMJ.* 2009;338:b2393.

32. Haukoos JS, Lewis RJ. The propensity score. *JAMA.* 2015;314:1637-1638.

47. Bogaerts JMK, Gussekloo J, de Jong-Schmit BEM, Le Cessie S, Mooijaart SP, van der Mast RC, Achterberg WP, Poortvliet RKE. Effects of the discontinuation of antihypertensive treatment on neuropsychiatric symptoms and quality of life in nursing home residents with dementia (DANTON): a multicentre, open-label, blinded-outcome, randomised controlled trial. *Age Ageing.* 2024;53:afae133.

65. Robins JM, Greenland S, Hu F-C. Estimation of the Causal Effect of a Time-Varying Exposure on the Marginal Mean of a Repeated Binary Outcome. *J Am Stat Assoc.* 1999; 94(447):687–700.

66. Daniel RM, Cousens SN, De Stavola BL, Kenward MG, Sterne JA. Methods for dealing with time-dependent confounding. *Stat Med.* 2013;32:1584-1618.

71. Brookhart MA, Wyss R, Layton JB, Sturmer T. Propensity score methods for confounding control in nonexperimental research. *Circ Cardiovasc Qual Outcomes.* 2013;6:604-611.

72. Albasri A, Hattle M, Koshiaris C, Dunnigan A, Paxton B, Fox SE, Smith M, Archer L, Levis B, Payne RA, Riley RD, Roberts N, Snell KIE, Lay-Flurrie S, Usher-Smith J, Stevens R, Hobbs FDR, McManus RJ, Sheppard JP, on behalf of the STRATIFY investigators. Association between antihypertensive treatment and adverse events: systematic review and meta-analysis. *BMJ.* 2021; 372: n189.

73. Vandenbroucke JP. When are observational studies as credible as randomised trials? *Lancet.* 2004;363:1728-1731.

a. Herrett E, Gallagher AM, Bhaskaran K, Forbes H, Mathur R, van Staa T, Smeeth L. Data Resource Profile: Clinical Practice Research Datalink (CPRD). *Int J Epidemiol.* 2015;44:827-836.
b. McGuinness LA, Warren-Gash C, Moorhouse LR, Thomas SL. The validity of dementia diagnoses in routinely collected electronic health records in the United Kingdom: A systematic review. *Pharmacoepidemiol Drug Saf.* 2019;28:244-255.
c. Morgan A, Sinnott SJ, Smeeth L, Minassian C, Quint J. Concordance in the recording of stroke across UK primary and secondary care datasets: a population-based cohort study. *BJGP Open.* 2021;5:BJGPO.2020.0117.
