# Supplementary material for: Associations between falls and other serious adverse events and antihypertensive medication in individuals with dementia: An observational cohort study
Source: PLoS Med. 2025 Sep 17;22(9):e1004731. doi: 10.1371/journal.pmed.1004731 (PMC12478963; doi:10.1371/journal.pmed.1004731)
Supplement: Attachment — Submitted filename: Response_to_Reviewers_R_auresp_3.docx [file pmed.1004731.s019.docx]

**Ms. #PMEDICINE-D-25-00036R2: “Associations between falls and other serious adverse events and antihypertensive medication in individuals with dementia: An observational cohort study” by Fujiwara et al.**

**Responses to the Editor and Reviewers**

We thank the Editor and Reviewers for their thoughtful comments and advice, which helped us to substantially improve our manuscript. We have responded to the Editors’ and Reviewers’ comments below. New text in the manuscript is highlighted in yellow.

**The academic editor:**

***Q. The rebuttal is comprehensive and compelling. Although I understand the author's reservations about the interpretation of figure S7, it could be included in the main text with the appropriate caveats.***

**Response:**

We thank the Academic Editor for highlighting the importance of formally testing for interaction between dementia status and antihypertensive treatment. We fully agree that stratified results alone are insufficient to infer effect modification, and that formal interaction terms are the appropriate statistical method.

As suggested, we have now incorporated the results of the interaction analyses (originally in Supplementary Table S7) into the main text and moved this table into the main manuscript as Table 2.

We also revised the Results section to state that statistical interaction between dementia status and treatment was observed in three out of four analytical approaches, though not in the inverse probability treatment weighting model.

Given our large sample size, we interpreted these findings with caution and now explicitly state that although interaction terms were statistically significant in most models, the magnitude of effect modification was small, and its clinical relevance may be limited. We have added these caveats to the Discussion to guide interpretation of these results.

**Page 30 Lines 495-499:**

Formal interaction tests between dementia status and antihypertensive treatment were statistically significant in three of the four analytical models, suggesting a modest but statistically detectable difference in relative risk by dementia status. However, the magnitude of effect modification was small, and given the large sample size, these findings should be interpreted cautiously as they may have limited clinical relevance.

| **Table 2. The association between the risk of hospitalisation or death from falls with antihypertensive treatment and dementia status in imputation datasets** | | | | | | | | | | | |
| --- | --- | --- | --- | --- | --- | --- | --- | --- | --- | --- | --- |
|  | **With dementia** | | | | | **Without dementia** | | | | |  |
|  | Exposure group | | Control group | |  | Exposure group | | Control group | |  | **P value for**  **interaction** |
|  | Population | Event | Population | Event | Hazard ratio (95% CI) | Population | Event | Population | Event | Hazard ratio (95% CI) |  |
| **Falls** (primary outcome) | | | | | | | | | | | |
| Propensity score adjustment | 4,062 | 1,533 | 19,448 | 6,781 | 1.15 (1.08 – 1.22) | 142,385 | 14,684 | 1,053,837 | 73,101 | 1.07 (1.05 – 1.10) | <0.001 |
| Multivariable adjustment | 4,062 | 1,533 | 19,448 | 6,781 | 1.12 (1.04 – 1.19) | 142,385 | 14,684 | 1,053,837 | 73,101 | 1.10 (1.08 – 1.12) | 0.034 |
| Propensity score matching | 3,795 | 1,442 | 3,795 | 1,396 | 1.12 (1.04 – 1.22) | 131,199 | 13,383 | 131,199 | 13,576 | 1.06 (1.03 – 1.08) | 0.043 |
| Propensity score IPTW | 4,062 | 1,533 | 19,448 | 6,781 | 1.18 (1.09 – 1.28) | 142,385 | 14,684 | 1,053,837 | 73,101 | 1.21 (1.18 – 1.25) | 0.599 |
| Exposure group and control group indicates with antihypertensive prescription and without antihypertensive prescription, respectively. CI indicates confidence interval; IPTW, inverse probability of treatment weighting. | | | | | | | | | | | |

| **Table 3. Hazard ratios of the initiation of antihypertensive medication drugs for each outcome in imputation datasets** | | | | | | | | | | |
| --- | --- | --- | --- | --- | --- | --- | --- | --- | --- | --- |
|  | **With dementia** | | | | | **Without dementia** | | | | |
|  | Exposure group | | Control group | |  | Exposure group | | Control group | |  |
|  | Population | Event | Population | Event | Hazard ratio (95%CI) | Population | Event | Population | Event | Hazard ratio (95%CI) |
| **Hypotension** | | | | | | | | | | |
| Propensity score adjustment | 4,062 | 258 | 19,448 | 747 | 1.51 (1.29 to 1.78) | 142,385 | 2,595 | 1,053,837 | 7,039 | 1.70 (1.60 to 1.79) |
| Multivariable adjustment | 4,062 | 258 | 19,448 | 747 | 1.48 (1.26 to 1.75) | 142,385 | 2,595 | 1,053,837 | 7,039 | 1.63 (1.54 to 1.72) |
| Propensity score matching | 3,800 | 230 | 3,800 | 169 | 1.52 (1.21 to 1.90) | 131,186 | 2,309 | 131,186 | 1,487 | 1.60 (1.48 to 1.72) |
| IPTW | 4,062 | 258 | 19,448 | 747 | 1.55 (1.29 to 1.86) | 142,385 | 2,595 | 1,053,837 | 7,039 | 1.83 (1.70 to 1.96) |
| **Syncope** | | | | | | | | | | |
| Propensity score adjustment | 4,062 | 173 | 19,448 | 687 | 1.34 (1.11 to 1.61) | 142,385 | 2,276 | 1,053,837 | 11,490 | 1.25 (1.18 to 1.31) |
| Multivariable adjustment | 4,062 | 173 | 19,448 | 687 | 1.27 (1.05 to 1.54) | 142,385 | 2,276 | 1,053,837 | 11,490 | 1.25 (1.18 to 1.31) |
| Propensity score matching | 3,792 | 166 | 3,792 | 129 | 1.36 (1.06 to 1.75) | 131,248 | 2,081 | 131,248 | 1,839 | 1.21 (1.12 to 1.31) |
| IPTW | 4,062 | 173 | 19,448 | 687 | 1.32 (1.06 to 1.64) | 142,385 | 2,276 | 1,053,837 | 11,490 | 1.36 (1.26 to 1.46) |
| **Fracture** | | | | | | | | | | |
| Propensity score adjustment | 4,062 | 728 | 19,448 | 3,397 | 1.05 (0.96 to 1.15) | 142,385 | 9,429 | 1,053,837 | 63,726 | 1.00 (0.98 to 1.02) |
| Multivariable adjustment | 4,062 | 728 | 19,448 | 3,397 | 1.04 (0.95 to 1.13) | 142,385 | 9,429 | 1,053,837 | 63,726 | 1.02 (0.99 to 1.04) |
| Propensity score matching | 3,788 | 676 | 3,788 | 686 | 1.05 (0.93 to 1.18) | 131,335 | 8,672 | 131,335 | 9,252 | 0.99 (0.96 to 1.03) |
| IPTW | 4,062 | 728 | 19,448 | 3,397 | 1.03 (0.92 to 1.15) | 142,385 | 9,429 | 1,053,837 | 63,726 | 1.08 (1.05 to 1.12) |
| Exposure group and control group indicates with antihypertensive prescription and without antihypertensive prescription, respectively. CI indicates confidence interval; IPTW, inverse probability treatment weighting. | | | | | | | | | | |

**Reviewer #2:**

***Q. Authors have adequately and thoroughly all raised issues. The only minor point I feel they should still modify is the comparison with metaanalysis of RCTs (lines 527-531). In fact, the metaanalysis included for comparison in their previous work (ref. 9) is indeed ref. 72, which therefore is not "another previous metaanalysis". Therefore I would simply state that the association between antihypertensive treatment and falls was greater than the estimate obtained in a meta-analysis of randomised controlled trials. In fact it is unsurprising that the results of this paper were similar to their previous work, which included the same individuals as the present study and actually shows the same difference. Auuhors might emphasize again that one likely reason, beyond residual confounding, is the already cited exclusion of frailer patients, including people with dementia, from RCTs.***

**Response:**

We thank the reviewer for this helpful clarification. We agree with the interpretation that the present study found a similar association between antihypertensive treatment and falls as previously reported in our earlier cohort study (Ref. 9), and that both observational studies differ somewhat from the findings of a meta-analysis of randomised controlled trials (Ref. 72), which showed no significant association. We have revised the manuscript accordingly to better reflect this comparison.

We now state that the observed difference between our real-world observational findings and those from RCTs may reflect both residual confounding and differences in study populations—particularly the exclusion of frailer individuals and people with dementia from clinical trials.

We agree that this divergence does not represent a methodological limitation per se, but rather highlights the complementary value of real-world data when examining populations underrepresented in randomised trials.

**Page 28 Lines 461-469:**

While our previous observational study, which included the same individuals as the present analysis, demonstrated a similar association between antihypertensive treatment and serious adverse events [9], a meta-analysis of randomised controlled trials reported no significant association between antihypertensive treatment and fall risk [49]. This discrepancy may be partly explained by differences in study population, particularly the exclusion of frailer individuals and people with dementia from many clinical trials, in contrast to our real-world cohort. It may also reflect the influence of residual confounding, such as confounding by indication, which is more likely to affect observational data [50].

**Reviewer #3:**

***Q1. I agree with reviewer 1 that the interaction tests in Table S7 are the key result if the main aim is to compare differences in risk of AE from anti-hypertensive agents according to dementia risk. Comparing the 'significance' of each the outcome-exposure relationship between no dementia/dementia patients is termed 'differences in nominal significance' which sharply increases Type I error. This can be seen in the overlapping 95% CI in the effect estimates between no dementia/dementia participants. The interactions tested in S7 are the appropriate approach to examining heterogeneity by dementia status.***

**Response:**

We thank the reviewer for this important comment and for highlighting the appropriate use of interaction terms to assess heterogeneity by dementia status. As noted by the Academic Editor, a similar concern was raised, and in response, we have revised the manuscript accordingly. Specifically, we have moved the interaction results from Supplementary Table S7 into the main manuscript as Table 2, and we now explicitly report that statistical interaction between dementia status and antihypertensive treatment was observed in three of the four analytical approaches.

However, we also note that the magnitude of interaction was modest, and that no statistically significant interaction was observed in the inverse probability treatment weighting model. We have therefore added appropriate caveats in Discussion sections to clarify that, while statistically significant, the effect modification by dementia status may not be clinically meaningful.

We hope these revisions address the reviewer’s concern and enhance the clarity and interpretability of our findings.

***Q2. It is possible to see overall similar SMDs between the groups after matching/weighting a PS where there is limited overlap or strong variation in distribution between the treated and non-treated groups. This is a relatively simple check (histograms of PS by treatment status) and should be included in a revised manuscript.***

**Response:**

We thank the reviewer for this insightful comment. As suggested, we have now included histograms showing the distribution of propensity scores separately for individuals with and without dementia. These figures demonstrate good overlap in the propensity score distributions between both populations (Supplementary Figure S3).

**Page 21 Lines 335-337:**

To further assess the appropriateness of propensity score methods, we examined the distribution of propensity scores in individuals with and without dementia (Supplementary Figure S3).

**Supplementary Figure S3.** Distribution of propensity scores by treatment status in individuals with and without dementia

***Q3. The details of how the 'tests for trend' were completed should be included in the methods - there is a huge amount of variation in what this term means in the literature.***

**Response:**

We thank the reviewer for this important comment. To clarify, the trend analyses were performed using a Cox proportional hazards model, where the number of antihypertensive medications (0, 1, 2, ≥3) was included as an ordered categorical variable. This variable was specified as an R ordered factor, allowing the model to assess whether there was a linear trend in the log hazard of the outcome across increasing levels of medication use. The model also adjusted for imputed propensity scores and prior history of the outcome. These details have now been added to the Methods section of the manuscript.

**Page 19 Lines 302-309:**

To assess whether there was a linear trend in the risk of adverse events across increasing numbers of antihypertensive medications, we additionally conducted a trend analysis by modelling the number of antihypertensive drugs as an ordered categorical variable in the Cox models. This approach allowed us to formally test for a linear increase in hazard with increasing medication count, after adjusting for imputed propensity scores and prior history of the outcome. Trend tests were conducted separately in individuals with and without dementia using pooled estimates from multiply imputed datasets.

**Reviewer #4:**

***Q1. Response to Q3: "Importantly, any differences in age structure between the dementia and non-dementia groups were accounted for in our analyses, where we adjusted for age in all models and further controlled for baseline cardiovascular and frailty-related comorbidities."***

***For this to work, the adjustments in the model would need to be very accurate. This is questionable, both because the relationship between e.g. age and the outcomes may be complex (non-linear), and because the authors did not adjust for time varying covariates, meaning that any incident comorbidity (which is more likely to occur in older patients) is not adjusted for. To alleviate this concern, I think a sensitivity analysis wherein individuals with dementia are more exactly matched to their age- and comorbidity peers, and subsequently assessing significant differences in the effects of antihypertensive drugs, would be prudent.***

**Response:**

We thank the reviewer for raising the concern regarding the potential impact of age on outcome incidence and the risk of residual confounding. As noted in our previous response, we included individuals aged 40 and older in the cohort to allow for generalizability across the adult population. However, we fully recognize the complex relationship between age and the outcomes of interest, which is why we employed multiple analytic strategies to ensure that potential confounding—age included—was robustly addressed.

Within each group (dementia and non-dementia), we applied four adjustment methods: multivariable regression, propensity score adjustment, inverse probability of treatment weighting, and propensity score matching using 1:1 nearest-neighbor matching with a caliper of 0.2. In the PSM analyses for both groups, age was well balanced, with standardized mean differences for age <0.1 (see Supplementary Table 6).

Since statistical adjustment methods aims to remove the effect of confounders, and given that (i) age was well balanced in the propensity score matched samples, and (ii) treatment effect estimates were consistent across all four methods, we are confident that age-related confounding has been effectively addressed in both groups, and that any residual bias due to age is likely to be minimal.

Given this, and the fact that propensity score matching balances not only age but also a wide range of covariates—and uses a widely accepted caliper threshold—we are not certain what additional benefit would be gained from exact matching on age alone, beyond what our existing approach already provides.

As clarified in the revised Methods section, we conducted propensity score estimation and matching separately within the dementia and non-dementia groups. This approach allowed for the construction of two internally comparable matched cohorts and ensured that age and comorbidity structures were balanced within each analysis.

**Page 17 Lines 258-263:**

To account for differences in baseline characteristics between two groups, we conducted propensity score analyses separately within individuals with and without dementia. In each of these two groups, we generated propensity scores in each imputation dataset using multivariable logistic regression with prescription of antihypertensive medication as the outcome, and all baseline covariates included (listed in the previous section and in Supplementary Table S4 and S5).

***Q2. Response to Q5: "We acknowledge the potential limitations in diagnostic accuracy and date-stamping in electronic health record databases. However, CPRD GOLD is a high-quality, widely validated UK primary care database, and previous validation studies have shown acceptable levels of diagnostic accuracy for chronic conditions, including cardiovascular disease and dementia.a,b,c Moreover, our analyses did not rely solely on diagnostic codes. We also incorporated prescription and measurement data, which are generally well recorded in CPRD. Notably, all issued prescriptions are automatically documented in the electronic health record, providing a reliable source of exposure information for phamacoepidemiological studies."***

***The provided references (a,b,c) report sensitivity ranges of about 75% (for stroke) or only reported "positive predictive value" for dementia. Assuming an average sensitivity of 70-80%, there is a good chance that both dementia and comorbidity diagnosis may be missed, which may affect results. I think this should be mentioned in the limitations.***

**Response:**

We thank the reviewer for this thoughtful comment. We agree that, despite the high quality and widespread validation of CPRD GOLD, limitations in diagnostic sensitivity—particularly for dementia and certain comorbidities—may result in under-ascertainment or misclassification. Although previous validation studies have shown acceptable diagnostic accuracy for these conditions (references 26, 67, 68), we have now added a statement in the limitations section acknowledging that such misclassification may have influenced the study findings.

**Page 31 Lines 514-519:**

Furthermore, although CPRD GOLD is a widely validated and high-quality UK primary care database, the accuracy and completeness of diagnostic coding for dementia and related comorbidities may be imperfect. Previous validation studies have demonstrated acceptable levels of diagnostic accuracy for these conditions; however, under-ascertainment or misclassification remains possible and may have influenced our study findings [26,67,68].

**References**

9. Sheppard JP, Koshiaris C, Stevens R, Lay-Flurrie S, Banerjee A, Bellows BK, Clegg A, Hobbs FDR, Payne RA, Swain S, Usher-Smith JA, McManus RJ. The association between antihypertensive treatment and serious adverse events by age and frailty: A cohort study. *PLoS Med.* 2023;20:e1004223.

26. Herrett E, Gallagher AM, Bhaskaran K, Forbes H, Mathur R, van Staa T, Smeeth L. Data resource profile: Clinical Practice Research Datalink (CPRD). *Int J Epidemiol.* 2015;44:827-836.

49. Albasri A, Hattle M, Koshiaris C, Dunnigan A, Paxton B, Fox SE, Smith M, Archer L, Levis B, Payne RA, Riley RD, Roberts N, Snell KIE, Lay-Flurrie S, Usher-Smith J, Stevens R, Hobbs FDR, McManus RJ, Sheppard JP, on behalf of the STRATIFY investigators. Association between antihypertensive treatment and adverse events: systematic review and meta-analysis. *BMJ.* 2021; 372: n189.

50. Vandenbroucke JP. When are observational studies as credible as randomised trials? *Lancet.* 2004;363:1728-1731.

67. McGuinness LA, Warren-Gash C, Moorhouse LR, Thomas SL. The validity of dementia diagnoses in routinely collected electronic health records in the United Kingdom: A systematic review. *Pharmacoepidemiol Drug Saf.* 2019;28:244-255.

68. Morgan A, Sinnott SJ, Smeeth L, Minassian C, Quint J. Concordance in the recording of stroke across UK primary and secondary care datasets: a population-based cohort study. *BJGP Open.* 2021;5:BJGPO.2020.0117.
