# Supplementary material for: Associations between falls and other serious adverse events and antihypertensive medication in individuals with dementia: An observational cohort study
Source: PLoS Med. 2025 Sep 17;22(9):e1004731. doi: 10.1371/journal.pmed.1004731 (PMC12478963; doi:10.1371/journal.pmed.1004731)
Supplement: Attachment — Submitted filename: Response to Editor_R.docx [file pmed.1004731.s020.docx]

**Ms. #PMEDICINE-D-25-00036R3: “Associations between falls and other serious adverse events and antihypertensive medication in individuals with dementia: An observational cohort study” by Fujiwara et al.**

**Responses to the Editors**

We thank the Editor for their thoughtful comments and advice, which helped us to substantially improve our manuscript. We have responded to the Editors’ comments below. New text in the manuscript is highlighted in yellow.

***GENERAL EDITORIAL REQUESTS
1. * We appreciate that you provide the text under 'summary boxes. However at PLOS we ask for a short, non-technical Author Summary of your research to make findings accessible to a wide audience that includes both scientists and non-scientists. The Author Summary should immediately follow the Abstract in your revised manuscript. This text is subject to editorial change and should be distinct from the scientific abstract. Ideally each sub-heading should contain 2-3 single sentence, concise bullet points containing the most salient points from your study. In the final bullet point of ‘What Do These Findings Mean?’ Please include the main limitations of the study in non-technical language.***

***2. In the author summary, in the final bullet point of 'What Do These Findings Mean?', please include the main limitations of the study in non-technical language.***

**Response:**
In accordance with the submission guidelines, we revised summary boxes as below.

**Page 7 Line 91 to Page 8 Line 115:**

**Why Was This Study Done?**

・Antihypertensive drugs have been shown to reduce cardiovascular disease morbidity and mortality among all age groups.

・Previous studies have demonstrated that antihypertensive treatment is associated with increased risk of serious adverse events, especially in older people and those with severe frailty.

・However, there is a lack of firm evidence regarding the harm associated with antihypertensive treatment in individuals with dementia.

**What Did the Researchers Do and Find?**

・This was a retrospective analysis using nationally representative UK general practice population between 1998 and 2018, from electronic health records.

・In a total of 1,219,732 individuals (23,510 with dementia), antihypertensive treatment was associated with an increased risk of hospitalisation or death from falls, hypotension, and syncope in both individuals with and without dementia.

・The absolute risk of harm with antihypertensive treatment was significantly higher in individuals with dementia.

**What Do These Findings Mean?**

・These findings suggests that clinicians, patients and their carers should consider the risk of serious adverse events when starting new antihypertensive medications for individuals with dementia.

・The main limitation of this work is the possibility of unmeasured confounding, and heterogeneity in dementia diagnoses based on coded entries in the electronic health record.

***3. Please confirm that your abstract complies with our requirements, including format (three sections: Background, Methods and Findings, and Conclusions) and providing all the information relevant to this study type*** [***https://journals.plos.org/plosmedicine/s/submission-guidelines#loc-abstract***](https://journals.plos.org/plosmedicine/s/submission-guidelines#loc-abstract) 

**Response:**

We have reviewed our abstract and confirmed that it complies with your requirements, including the prescribed format and the inclusion of all information relevant to this study type.

***4. Please ensure that all abbreviations are defined at first use throughout the text.***

**Response:**

We have reviewed that all abbreviations were defined at first use throughout the text.

***5. Please confirm that all numbers presented in the abstract are present and identical to numbers presented in the main manuscript text.***

**Response:**

We have confirmed that all numbers presented in the abstract are present and identical to numbers presented in the main manuscript text.

***GENERAL
6. Please review your text for claims of novelty or primacy (e.g. 'for the first time') and remove this language. In addition, please check that any use of statistical terms (such as trend or significant) are supported by the data, and if not please remove them.***

**Response:**

In accordance with the submission guidelines, we have revised the relevant parts.

**Page 25 Lines 410-419:**

In this nationwide electronic health record data-based observational study of 1.2 million previously untreated individuals, antihypertensive treatment was associated with an increased risk of hospitalisation or death from falls, hypotension, and syncope in both individuals with and without dementia, with comparable relative risks observed between the two groups. However, the absolute risk of harm with antihypertensive treatment was significantly higher in individuals with dementia than in those without dementia. These findings indicate that the absolute risk of serious adverse events with antihypertensive drugs differs by dementia status, and that careful consideration of the priorities of individuals with dementia is necessary when starting new antihypertensive medications in this group.

**Page 30 Lines 510-512:**

Strengths of this study include the large general practice population-based sample and the examination of how the association between antihypertensive therapy and serious adverse events differ by dementia status.

***7. Please remove the 'conclusions' subheading from the discussion. Please also remove any other subheadings from the discussion.***

**Response:**

We have removed all subheadings from the discussion, including the 'conclusions'.

**Page 34 Line 589 to Page 35 Line 595:**

Taken together, in previously untreated individuals with elevated systolic BP, this study demonstrated the increased absolute risk of serious adverse events associated with antihypertensive treatment in individuals with dementia compared to those without dementia. When prescribing antihypertensive medication for individuals with dementia, careful consideration of potential adverse events is needed. Clinicians, patients and their carers should carefully consider the balance between benefits and harms of antihypertensive treatment for individuals with dementia.

***8. Statistical reporting: Please revise throughout the manuscript, including tables and figures.
- Please report statistical information as follows to improve clarity for the reader ""22% (95% CI [13,28]; p</=)"".
- Please separate upper and lower bounds with commas instead of hyphens as the latter can be confused with reporting of negative values.
- Please repeat statistical definitions (HR, CI etc.) for each set of parentheses.***

**Response:**

In accordance with the submission guidelines, we have repeated the statistical definitions within each set of parentheses for clarity, and have separated the upper and lower bounds of the 95% confidence intervals with “to.”

***9. In the last sentence of the Abstract Methods and Findings section, please describe the main limitation(s) of the study's methodology.***

**Response:**

In accordance with the submission guidelines, we described the main limitations of this study.

**Page 4 Line 52 to Page 5 Line 83:**

**Methods and Findings:** This was a retrospective analysis using nationally representative UK general practice population between 1998 and 2018, from electronic health records (Clinical Practice Research Datalink, CPRD, GOLD). Eligible individuals were aged ≥40 years, with a systolic blood pressure 130–179 mmHg, and not previously prescribed antihypertensive treatment. The diagnosis of dementia was based on clinical codes in the electronic health record. Individuals were allocated to the exposure group if they were prescribed at least one antihypertensive medication during a 12-month exposure period. Those who were not prescribed any antihypertensive medication during the exposure period were allocated to the control group. The primary outcome was the first hospitalisation or death from a fall within 10 years of the follow-up period. Secondary outcomes were first hospitalisation or death from hypotension, syncope, and fracture. In a population of 1,219,732 individuals, 23,510 had dementia. Antihypertensive medications were newly prescribed in 4,062/23,510 (17.3%) individuals with dementia and 142,385/1,196,222 (11.9%) individuals without dementia in the 12 months exposure period. In the primary analyses, which adjusted for the propensity score and a previous history of the outcome of interest, antihypertensive treatments were associated with a small increased risk of hospitalisation or death from falls (adjusted hazard ratio [aHR] 1.15, 95% confidence interval [CI] 1.08 to 1.22), hypotension (aHR 1.51, 95%CI 1.29 to 1.78), syncope (aHR 1.34, 95%CI 1.11 to 1.61), but not fracture (aHR 1.05, 95%CI 0.96 to 1.15), in individuals with dementia. These findings were consistent across different analytic approaches, including multivariable adjustment, propensity score matching, and inverse probability treatment weighting. In individuals without dementia, the association between antihypertensive treatment and serious adverse events was similar, with a small increased risk of hospitalisation or death from falls (aHR 1.07, 95%CI 1.05 to 1.10). However, the absolute fall risk associated with antihypertensive treatment was significantly higher in individuals with dementia (47 per 10,000 individuals per year, 95%CI 26 to 70) compared to those without (14 per 10,000 individuals per year, 95%CI 10 to 18). The absolute risks of hypotension and syncope with antihypertensive treatment were also higher in the individuals with dementia compared to those without. The main limitation is the possibility of unmeasured confounding, and heterogeneity in dementia diagnoses based on coded entries in the electronic health record.

***10. In the abstract, please include the important dependent variables that are adjusted for in the analyses.***

**Response:**

We have added a more specific description of the independent variables used in the multivariable analyses to the abstract. However, due to the word limit, it was not possible to include all independent variables used in generating the propensity score.

**Page 5 Lines 66-71:**

In the primary analyses, which adjusted for the propensity score and a previous history of the outcome of interest, antihypertensive treatments were associated with a small increased risk of hospitalisation or death from falls (adjusted hazard ratio [aHR] 1.15, 95% confidence interval [CI] 1.08 to 1.22), hypotension (aHR 1.51, 95%CI 1.29 to 1.78), syncope (aHR 1.34, 95%CI 1.11 to 1.61), but not fracture (aHR 1.05, 95%CI 0.96 to 1.15), in individuals with dementia.

***FUNDING STATEMENT
11. The funding statement should include URLs of the funding agencies.***

**Response:**

In accordance with the submission guidelines, we added URLs of the funding agencies in the funding statement.

**Page 37 Lines 606-616:**

**Sources of Funding**

TF received funding from the SENSHIN Medical Research Foundation (https://www.smrf.or.jp/). JS and CK receive funding from the Wellcome Trust/Royal Society via a Sir Henry Dale Fellowship (ref: 211182/Z/18/Z) (https://www2.fundsforngos.org/fellowships/wellcome-trust-seeking-applications-for-henry-dale-fellowships/). This research was funded in part, by the Wellcome Trust (ref: 211182/Z/18/Z) (https://wellcome.org/). FDRH is part supported by the NIHR Applied Research Collaboration (ARC) Oxford Thames Valley (https://www.arc-oxtv.nihr.ac.uk/). RJM is supported by NIHR Oxford and Thames Valley Applied Research Consortium (https://www.arc-oxtv.nihr.ac.uk/). The funders had no role in study design, data collection and analysis, decision to publish, or preparation of the manuscript.

***COMPETING INTERESTS STATEMENT
12. All authors must declare their relevant competing interests per the PLOS policy, which can be seen here:*** [***https://journals.plos.org/plosmedicine/s/competing-interests***](https://journals.plos.org/plosmedicine/s/competing-interests) ***For authors with ties to industry, please indicate whether any of the interests has a financial stake in the results of the current study.***

**Response:**

We have declared that no competing interests exist for this study.

**Page 2 Line 31:**

**Competing interests:** The authors have declared that no competing interests exist.

***DATA AVAILABILITY
13. Please provide an URL or an email address for the CPRD.***

**Response:**

The URL of the CPRD has been provided in the manuscript.

**Page 11 Lines 174 to 175:**

Data were obtained via a CPRD institutional licence. Requests for data sharing should be made directly to the CPRD ([https://cprd.com](https://cprd.com/)).

***14. Please include the links to NHS digital and the office for national statistics (which are mentioned in the data availability in the methods section), in the Data availability statement in the meta-data.***

**Response:**

The URL of the NHS Digital has been provided in the manuscript.

**Page 11 Line 175 to Page 12 Line 177:**

The Hospital Episode Statistics data used in this analysis are re-used with permission from NHS Digital ([https://digital.nhs.uk](https://digital.nhs.uk/)) who retain the copyright for that data.

***FIGURES
15. When a p value is given, please specify the statistical test used to determine it in the legend.***

**Response:**

We specified that we used the log-rank test for the statistical test in Figure 1.

**Page 52 Lines 852 to 859:**

**Fig. 1 Cumulative risk of serious adverse events by antihypertensive medication according to categories of dementia and drug exposure status.**

Kaplan-Meier curves of the cumulative incidence of serious adverse events by the four categorical groups are shown. Figure 1A, 1B, 1C and 1D shows the cumulative incidence of falls, hypotension, syncope and fracture, respectively. Each solid line indicates cumulative incident rate for each serious adverse event and its around area indicates the 95% confidence interval. For all p values reported in the figures, the statistical test used was the log-rank test.

***16. Please consider avoiding the use of red and green in order to make your figure more accessible.***

**Response:**

We appreciate the suggestion to adjust the color scheme to improve accessibility. However, as the first author no longer has access to the date drive in University of Oxford, we are unable to regenerate the figures using an alternative color palette. We therefore respectfully request to retain the current color scheme in the submitted figures.

***OBSERVATIONAL, COHORT, CROSS-SECTIONAL, AND CASE CONTROL STUDIES
17. Did your study have a prospective protocol or analysis plan? Please state this (either way) early in the Methods section.***

***a) If a prospective analysis plan (from your funding proposal, IRB or other ethics committee submission, study protocol, or other planning document written before analyzing the data) was used in designing the study, please include the relevant prospectively written document with your revised manuscript as a Supporting Information file to be published alongside your study, and cite it in the Methods section. A legend for this file should be included at the end of your manuscript.

b) If no such document exists, please make sure that the Methods section transparently describes when analyses were planned, and when/why any data-driven changes to analyses took place.

c) In either case, changes in the analysis-- including those made in response to peer review comments-- should be identified as such in the Methods section of the paper, with rationale.***

**Response:**

This was a retrospective observational cohort study.

***RESULTS
18. Please consider stating the primary and secondary outcomes in the subheaders, rather than just ‘primary outcome’ for ease of the reader.***

**Response:**

We have already presented the primary and secondary outcomes in separate sections, which we believe makes them clear and easy for the reader to follow.
